# Sorcin Links Calcium Signaling to Vesicle Trafficking, Regulates Polo-Like Kinase 1 and Is Necessary for Mitosis

**DOI:** 10.1371/journal.pone.0085438

**Published:** 2014-01-10

**Authors:** Vasiliki S. Lalioti, Andrea Ilari, David J. O'Connell, Elena Poser, Ignacio V. Sandoval, Gianni Colotti

**Affiliations:** 1 Centro de Biología Molecular Severo Ochoa, CSIC -Universidad Autónoma de Madrid, Departamento Biología Celular e Inmunología, Cantoblanco; Centro de Investigación Biomédica en Red de Enfermedades Hepáticas y Digestivas (CIBERehd), Madrid, Spain; 2 CNR-National Research Council of Italy, Institute of Molecular Biology and Pathology c/o Department of Biochemical Sciences “A. Rossi Fanelli”, University “Sapienza” P.le A.Moro 5, Rome, Italy; 3 Conway Institute of Biomolecular & Biomedical Research, University College Dublin, Dublin, Ireland; 4 Department of Biochemical Sciences “A. Rossi Fanelli”, University “Sapienza” P.le A.Moro 5, Rome, Italy; National Cancer Institute, NIH, United States of America

## Abstract

Sorcin, a protein overexpressed in many multi-drug resistant cancers, dynamically localizes to distinct subcellular sites in 3T3-L1 fibroblasts during cell-cycle progression. During interphase sorcin is in the nucleus, in the plasma membrane, in endoplasmic reticulum (ER) cisternae, and in ER-derived vesicles localized along the microtubules. These vesicles are positive to RyR, SERCA, calreticulin and Rab10. At the beginning of mitosis, sorcin-containing vesicles associate with the mitotic spindle, and during telophase are concentrated in the cleavage furrow and, subsequently, in the midbody. Sorcin regulates dimensions and calcium load of the ER vesicles by inhibiting RYR and activating SERCA. Analysis of sorcin interactome reveals calcium-dependent interactions with many proteins, including Polo-like kinase 1 (PLK1), Aurora A and Aurora B kinases. Sorcin interacts physically with PLK1, is phosphorylated by PLK1 and induces PLK1 autophosphorylation, thereby regulating kinase activity. Knockdown of sorcin results in major defects in mitosis and cytokinesis, increase in the number of rounded polynucleated cells, blockage of cell progression in G2/M, apoptosis and cell death. Sorcin regulates calcium homeostasis and is necessary for the activation of mitosis and cytokinesis.

## Introduction

The role of calcium and calcium binding proteins in mitosis is not completely understood, although there is general agreement for their involvement in cell cycle regulation [1]. Studies in several cell types revealed a peak in free Ca^2+^ concentration during mitosis between metaphase and cytokinesis [2], [3]. In addition, Kao et al. (1990) showed that Ca^2+^ transients precede and are always strictly correlated with nuclear envelope breakdown and metaphase-anaphase transition in 3T3 fibroblasts [4]. Changes of free Ca^2+^ concentrations induce activation of protein kinase cascades in the cytoplasm including CaMKs, PKA, MAPKs resulting in the translocation of transcription factors such as nuclear factor kappa-light chain-enhancer of activated B cells (NFκB) and nuclear factor of activated T-cells (NFAT) from the cytosol to the nucleus, where they switch on transcription of specific genes, related to inflammatory or immune responses, cell survival responses or cell proliferation [1], [5]. In addition, EF-hand proteins such as calmodulin and calcineurin are important calcium sensor proteins and cell cycle regulators [6], [7].

Sorcin (soluble resistance-related calcium-binding protein) is a 21.6-kDa calcium-binding protein belonging to the penta-EF-hand (PEF) family which includes also calpains, grancalcin, ALG-2, peflin and PEF1. Sorcin has a two-domain architecture, characterized by a flexible and hydrophobic Gly/Pro-rich N-terminal domain and a C-terminal calcium-binding domain containing the five EF-hand motifs, and dimerizes through the unpaired EF5 hand. Ca^2+^ binding to sorcin increases the exposure of hydrophobic surfaces and triggers the reversible translocation of sorcin from the cytoplasm to cell membranes, where it interacts with specific target proteins and participates in a variety of physiological processes. Sorcin is expressed in a wide set of human cell types, such as cardiac cells, vascular smooth muscle cells and adrenal medulla, and participates in the regulation of a variety of cell-specific calcium-dependent functions. In cardiac and smooth muscle cells, sorcin interacts in a Ca^2+^-dependent fashion with the ryanodine receptor 2 (RYR2), the Na^+^-Ca^2+^ exchanger NCX1, the L-type voltage-dependent Ca^2+^ channel and sarcoplasmic/endoplasmic reticulum Ca^2+^-ATPase (SERCA), and regulates these proteins [8]–[12], thus modulating calcium-induced calcium release. In the adrenal medulla, sorcin binds to synexin (Annexin 7) and inhibits synexin-mediated calcium-dependent chromaffin granule aggregation.

In addition, sorcin is overexpressed in many human tumors, such as leukemia, gastric, breast and ovarian cancers [13]–[17]. Sorcin is found to be highly expressed in chemoresistant cell lines [18]–[24], its overexpression confers Multi Drug Resistance (MDR), and treatment with antisense oligonucleotides increases cell sensitivity for vincristine and other antitumor drugs [25], [26], suggesting that sorcin might be a useful marker of MDR and a therapeutic target for reversing tumor multidrug resistance.

To gain insights about novel functional roles of sorcin we performed a comprehensive analysis of the sorcin interactome and subcellular localization and performed biochemical and knockdown studies. In particular, we have investigated the role of sorcin during the cell cycle of 3T3-L1 fibroblasts, the established model of the most common cells of connective tissue. Using fluorescence microscopy, we have established that sorcin is located in the nucleus, in cytosolic ER vesicles and macrovesicles localized along the microtubules, in the plasma membrane and in the midbody. Cell fractionation, immunoprecipitation, in vitro protein-protein interaction arrays, enzyme activity assays and surface plasmon resonance experiments demonstrated that sorcin is able to establish calcium-dependent interactions with many protein targets. We demonstrated that sorcin is phosphorylated by PLK1 and induces PLK1 autophosphorylation, thereby regulating PLK1 kinase activity. Knock down of sorcin affects mitosis and cytokinesis in a pathway that involves PLK1.

## Materials and Methods

### Cloning, mutagenesis, transformation, expression, protein purification

The cDNA of human sorcin (I.M.A.G.E. Consortium clone 4281626) was subcloned in the pCDNA3.1+ vector (Invitrogen). The following oligonucleotides were used for human sorcin gene amplification: SHForHind: 5′-GAAGCTTGCAGCATGGCGTACCC-3′ and SHRevXho: 5′-CCTCTCGAGTTAAACACTCATGACAC-3′. The amplification products obtained were digested with XhoI and HindIII and cloned in the pCDNA3.1+ vector previously treated with HindIII and XhoI.

GFP-sorcin was cloned in the pCDNA3.1+ vector, using the same amplification product, treated with HindIII and XhoI, which was joined with the amplification product of the EGFP gene, obtained from the plasmid pEGFP-N1 (Clontech) using the primers GFPKpnFor: 5′-GATCCACCGGGGTACCCCATGGTG-3′, and GFPHindRev: 5′- GAGCTGTACAAAGCTTGCGGCCGCG-3′.

Sorcin A2C, S114D, S117D, S119D, SS149/150DD, T155D mutants were obtained by site-directed mutagenesis using the QuikchangeII kit (Stratagene) using the PfuUltra polymerase, from the pET22b-human wt-sorcin expression vector (Novagen, Madison, WI, USA) previously described [10].

The oligonucleotides used were:

SorC2FOR: 5′- GAAGGAGATATACCATGTGCTACCCGGGGCATCCTGG-3′,

SorC2REV: 5′- CCAGGATGCCCCGGGTAGCACATGGTATATCTCCTTC- 3′,

SorD114FOR: 5′-GACAACACTTTATCAGTTTTGACGATGACAGGAGTGGAACAGTAGAC-3′,

SorD114REV: 5′-GTCTACTGTTCCACTCCTGTCATCGTCAAAACTGATAAAGTGTTGTC-3′,

SorD117FOR: 5′-CAGTTTTGACACTGACAGGGATGGAACAGTAGACCCACAAG-3′,

SorD117REV: 5′-CTTGTGGGTCTACTGTTCCATCCCTGTCAGTGTCAAAACTG-3′,

SorD119FOR: 5′-GACACTGACAGGAGTGGAGATGTAGACCCACAAGAATTGCAG-3′,

SorD119REV: 5′-CTGCAATTCTTGTGGGTCTACATCTCCACTCCTGTCAGTGTC-3′,

SorD149150FOR: 5′-GTGAATTCAATTGCAAAACGATACGATGATAATGGAAAGATCACCTTCG-3′,

SorD149150REV: 5′-CGAAGGTGATCTTTCCATTATCATCGTATCGTTTTGCAATTGAATTCAC-3′,

SorD155FOR: 5′-CAGCACCAATGGAAAGATCGATTTCGACGACTACATCGCC-3′,

SorD155REV: 5′-GGCGATGTAGTCGTCGAAATCGATCTTTCCATTGGTGCTG-3′,

The sequences of the mutated vectors were verified by the Sanger dideoxynucleotide technique. The recombinant plasmid was expressed in *E. coli* BL21(DE3) cells.

Wt-sorcin, hamster sorcin, the sorcin calcium binding domain (HSBD) and sorcin mutants were expressed in *E. coli* BL21(DE3) cells, and purified according to Meyers et al. [12].

AlexaFluor 532 C_5_-maleimide (Life technologies) selectively reacts with thiol groups exposed on the surface of proteins. In order to conjugate the mutant sorcin and the probe, a volume of 4 ml of A2C sorcin (2 mg/ml in 20 mM Hepes, pH 7.2) was exposed to an excess of reducing agent (5 mM dithiothreitol) that was then removed using a G25 column equilibrated in 20 mM Hepes, pH 7.2. Subsequently, 1 ml of reactive dye solution of 10 mM AlexaFluor 532 C_5_-maleimide dissolved in DMSO was added to the protein solution. The reaction proceeded for 8 hours at 4°C; the excess of dye was removed using a gel filtration column equilibrated in 20 mM Hepes, pH 7.2. All procedures including the dye were performed in the dark.

### Protein array experiments

The ProtoArray (Life technologies) human protein microarray contains over 9000 unique human full length proteins individually purified and arrayed in duplicate on a nitrocellulose coated glass slide under native conditions, to maximize their functionality. Protein array screening experiments were carried out according to the procedures reported in the ProtoArray application guide, using 1 µM AlexaFluor 532-A2C sorcin in the presence of either 1 mM EDTA or 1 mM CaCl_2_.

### Cell cultures and transfections

Normal rat kidney (NRK) cells, mouse 3T3-L1 fibroblasts (American Type Culture Collection), 293FT (Invitrogen, Life Technologies), African green monkey kidney fibroblasts (Cos7) and human hepatoma cellular carcinoma cells Huh7 (kindly donated by Dr. Esteban Dominco, CBM Madrid) [27] were grown on plastic dishes or 10-mm glass coverslips using Dulbecco's modified Eagle's medium supplemented with 10% foetal calf serum, 4 mM glutamine, 50 mg/l streptomycin, 100 IU/l penicillin and non-essential amino acids at 37°C in a humidified CO_2_ incubator. 3T3-L1 adipocytes were differentiated from 3T3-L1 fibroblasts as previously described [28].

Transient cDNA transfections were performed using lipofectamine 2000; the transfected cells were additionally incubated 3 h with 10 μg/ml cycloheximide.

### Fura2-AM Ca^2+^ ratio measurement

Cos7 cells grown on 0.17 mm glass bottom microplates and transfected with GFPsorcin for 15 h were incubated in cell medium containing 2 mM Fura2-acetoxymethyl ester (Fura2-AM, Molecular Probes) and 0.02% non-ionic detergent Pluronic F-127 for 1 h under normal culture conditions and 30 min longer with normal cell medium. For imaging experiments, cells were kept under controlled O_2_/CO_2_ conditions and images were collected in a Camera Andor 885EM using excitation wavelengths of 340 and 380 nm. Fluorescence was detected at 535 nm, and ratios (340/380) were assessed using the Leica Application suite 2.2.2 build 4842 software [29].

### Antibodies

The mouse α-sorcin (33–800) was from Zymed, the rabbit α-Annexin 11 (NB100-78588) was from Novus Biologicals, the rabbit α-Caveolin (610060) from BD Transduction Laboratories, the mouse α-actin (JLAA20), the mouse α-Na/K ATPase(a5) from the University of Iowa, the mouse α-Ryr (MA3-925) from Antibody Directory, the mouse α-EEA1 (610457) from BD Transduction Laboratories, the rabbit α-Polo-like kinase 1 (H-152) sc-5585 and the rat α-YL1/2 tubulin (sc-53029) from Santa Cruz, the rabbit α-Rab8 (D22D8), α-Rab7 (D95F2) and α-Rab10 (D36C4) from Cell Signaling, the rabbit α-Rab11 (715300) from Invitrogen, the human α-Ubf was gift from M.M. Valdivia (Universidad de Cadiz), the rabbit α-PDI was gift from J.G. Castaño (IIB Universidad Autonoma Madrid) the mouse α-Nuclear pore complex proteins (mad 414 NUP) from Sigma, the polyclonal rabbit α-sorcin and the p30 chromatin protein antibodies were developed in the laboratory [30], [31].

### Conventional and confocal light microscopy studies

For immunouorescence staining, cells were plated and grown on 10 mm glass coverslips, fixed with 2% paraformaldehyde for 20 min, permeabilized with 0.2% Triton X-100 for 10 min and incubated in 50 mM glycine for 30 min more. Primary antibody dissolved in 1% bovine serum albumin was added and allowed to incubate overnight at 40°C. Primary antibody was removed, wells washed and secondary AlexaFluor 488, 594 or 647 was added and incubated for 1 h at room temperature. Conventional immunofluorescence and confocal microscopy were performed using an Axiovert135M microscope (Zeiss) and the confocal Zeiss multiphoton LSM710 inverted microscope. Following lentiviral treatments, 3T3-L1 fibroblasts were imaged for cell morphology under bright field conditions using an Axiovert135 Zeiss inverted microscopy with a monochromic ccd camera. The Fiji image processing package was used for imaging of all microscopy studies, and in particular for size measurements of the cytosolic vesicles.

### Lentiviral production and transduction

A lentiviral system based on tTRKRAB repressor was used to obtain sorcin silencing in 3T3-L1 fibroblasts. The lentivectors pMD2G (encoding the VSV-G envelope protein), psPAX2 (packaging vector), and the pLVTHM (cloning vector) were from Trono Laboratory and were purchased from Addgene.

The following oligonucleotides were synthesized and used for the generation of shRNA:

1F182: 5′-CGCGTCCCCGGACGGACAAATTGATGCTTTCAAGAGACCTGCCTGTTTAACTACGATTTTTGGAAAT-3′,

1R182: 5′-CGATTTCCAAAAAGGACGGACAAATTGATGCTTCTCTTGAACCTGCCTGTTTAACTACGAGGGGA-3′,

2F231: 5′-CGCGTCCCCCACAGTCTGGCATTGCGGGTTCAAGAGACCCGCAATGCCAGACTGTGTTTTTGGAAAT-3′,

2R231: 5′-CGATTTCCAAAAACACAGTCTGGCATTGCGGGTCTCTTGAACCCGCAATGCCAGACTGTGGGGGA-3′.

The cloning of the silencing oligonucleotides in the pLVTHM vector, the generation of infectant viruses and the use of GFP marker for the expression quantification in 3T3-L1 fibroblasts were carried out using the protocol of Szulc and Aebisher [32]. Cell media containing shRNA lentivirus was collected 48 and 72 hours after initial transfection, filtered and concentrated by centrifugation 120 min at 50.000 g, 16°C. The transduction of 40% confluent 3T3-L1 fibroblasts was made overnight with 10^−3^ concentrated lentivirus treated before with 6 ug/ml polybrene for 3 hours. The gene silencing efficacy of siRNAs was measured by densitometry quantifications of Western Blots with Quantity 1-D software, using the Biorad GS-800 calibrated densitometer, and the Na-ATPase as a reference.

### Fractionation of cultured cells

Cultured cells were washed twice in cold PBS, resuspended in cold buffer A (20 mM HEPES, pH 7.4, 0.25 M sucrose, 1 mM EDTA, 50 mM β-glycerophosphate, 50 mM NaF, 1 mM NaVO_4_, 1 mM phenylmethylsulfonyl fluoride, 5 µg/ml leupeptin, 5 µg/ml aprotinin, and 1.5 µM pepstatin), disrupted by flushing through a 23-G needle 20 times and centrifuged for 10 min at 1000 g. The nuclei-ER contained pellet was solubilized by treatment for 1 min with 1% NP-40 in buffer A at 4°C and the ER proteins were recovered in the supernatant by centrifugation for 6 min at 1000 g. Highly purified nuclei were prepared [33], [34]. Postnuclear membranes were collected by centrifugation for 90 min at 150000 g. The fractionation of 3T3-L1 fibroblasts in low density microsomes (LDM), high density microsomes (HDM), cytosol and plasma membrane was performed as described [35]. Pellets of cellular fractions resuspended in buffer A and cytosol were boiled for 10 min in Laemmli buffer, subjected to SDS-PAGE, and blotted onto nitrocellulose, and sorcin was studied by western blotting.

### Cell Lysis, Immunoprecipitation, and Western Analysis

3T3-L1 fibroblasts were lysed in buffer A containing 1% Nonidet P-40, centrifuged for 10 min 15000 g and the supernatant was used for immunoprecipitation experiments by incubating for 3 h at 4°C with the corresponding antibodies. The protein-antibody complexes were collected on protein G-Sepharose and washed four times with buffer A containing 0.5% Nonidet P-40 and once with 0.1% SDS in buffer A.

### In vitro Polo-like kinase 1 assay

PLK1 assay was carried out with purified recombinant PLK1 according to manufacturer's protocol (Cell Signaling Technology, #7728), using purified β-casein (Sigma C6905) as control PLK1 target. Briefly, 5 μg of each substrate was incubated with 0.4 μg of PLK1 in the presence of 5 μl 0.16 μCi/μl [^32^P]ATP solution for 15 min at 30°C. The reactions were stopped by addition of Laemmli sample buffer and analyzed by SDS-PAGE. P^32^-labeled protein bands in autoradiographic images were quantified with Quantity 1-D software using the Biorad GS-800 calibrated densitometer.

### Cell-cycle analysis

Following treatments, cells were trypsinized, washed with PBS and fixed in ice cold 100% ethanol. Cells were then washed with PBS, stained with propidium iodide (Sigma) and analyzed on a cytometer FACScalibur111 Becton Dickinson. Cell-cycle distribution was analyzed using CellQuestPro software.

## Results

### Sorcin interactions *in vitro*: protein array experiments

Through protein array experiments we identified 224 proteins which bind sorcin (Figure S1). Among these, 222 proteins are not reported to bind to sorcin, and only Annexin 11 and the voltage-dependent calcium channel subunit CACNB1 are known sorcin interactors (Table S1). Most of these interactions are calcium-dependent, since in the presence of 1 mM calcium the interactions with sorcin take place with an average of 1.84 higher level of signal (full range: 0.5–4.0 times) with respect to the same experiments carried out in the presence of 1 mM EDTA, and more than 90% of the interactions are increased or decreased by at least 30% in the presence of calcium (Table S1). Many proteins in the list of sorcin interactome possess one or more hydrophobic sequences rich in glycine, tyrosine and proline residues, which have been identified to interact with sorcin in the case of sorcin-annexin VII interaction [36], [37] (data not shown). The 224 proteins have been categorized with the help of the Bioprofiling and Cytoscape softwares, with respect to the molecular functions sorcin may be involved in, according to Gene Ontology categories (Tables S1 and S2).

Strikingly, among the interactors of sorcin we found several components of the spindle and of the midbody (Tables S2, S3). The odd ratio (i.e. the ratio of occurrence for GO term in the input list to the occurrence for GO term in the whole genome; average odd ratio is 1) for the midbody proteins is 6.7, according to Bioprofiling analysis. Among the kinases that interact with sorcin, the serine/threonine kinases are prevalent, as 15 members of this group interacted with sorcin (Table S2), and PLK1, Aurora A, Aurora B, CSNK2A1 and CSNK2A2 were cell cycle regulators [38].

### Sorcin expression and localization

**Figure 1:**
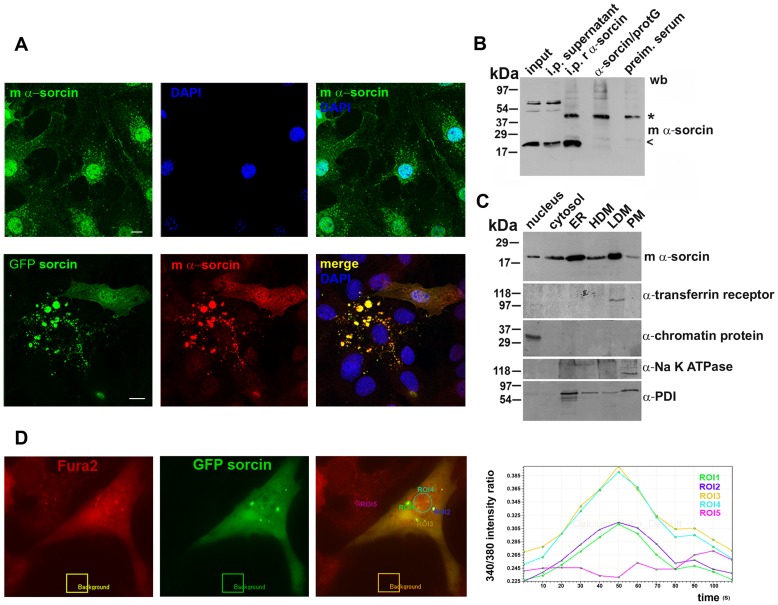
Cellular distribution of sorcin. A: 3T3-L1 fibroblasts were paraformaldehyde-fixed, permeabilized with Triton X-100, stained using sorcin-specific mouse monoclonal antibodies, DNA-stained using DAPI. 3T3-L1 cells were transfected with GFPsorcin for 15 h, incubated 3 h with 10 µg/ml cycloheximide, and stained with mouse monoclonal sorcin-specific antibodies. Sorcin was detected in the nucleus, cytoplasmic vesicles and plasma membrane. Ectopically expressed sorcin massively accumulated in the macrovesicles, nucleus and plasma membrane. *Bars 10 µm*. B: Western blot of input fraction (input), supernatant of immunoprecipitation with rabbit polyclonal α-sorcin (i.p. supernatant), immunoprecipitation with rabbit polyclonal α-sorcin (i.p. r α-sorcin), rabbit polyclonal α-sorcin bound to protein G (α-sorcin/protG) and pull down with the preimmune serum (preim. serum). Samples analysed by SDS-PAGE and western blot using monoclonal mouse antibodies against sorcin. C: Cellular fractionation of 3T3-L1 fibroblasts into nuclei, cytosol, high-density microsomes (HDM), low-density microsomes (LDM), and plasma membrane (PM). One fifth of the cytosol fraction and one tenth of each of the other fractions were resolved by SDS-PAGE and analyzed by western blot using specific antibodies against sorcin, transferrin receptor, chromatin protein, Na^+^-K^+^ ATPase and PDI. High amount of sorcin is accumulated mainly in ER and in LDM, but a significant amount appears specifically in the nucleus. D: Upon ectopic expression, ER-derived sorcin vesicles contain high amounts of calcium. Cos7 cells transfected for 15 h with GFPsorcin were exposed to Fura2AM for 1 h in normal culture conditions. GFPsorcin vesicles perfectly colocalize with the vesicles loaded with Fura2AM. ROI1, ROI2 and ROI3 surround GFPsorcin vesicles, ROI4 the nucleus and ROI5 Fura-loaded vesicles in cell without ectopic expression. An empty area outside of the cell was used to perform a background subtraction. The graph depicts the change in the 340/380 intensity ratio over time.

We found high sorcin expression in fibroblasts, hepatocarcinoma Huh7, 3T3-L1 adipocytes, normal rat kidney epithelial cells (NRK) and human 293FT embryonic kidney cells (Figures 1, S2).

**Figure 2:**
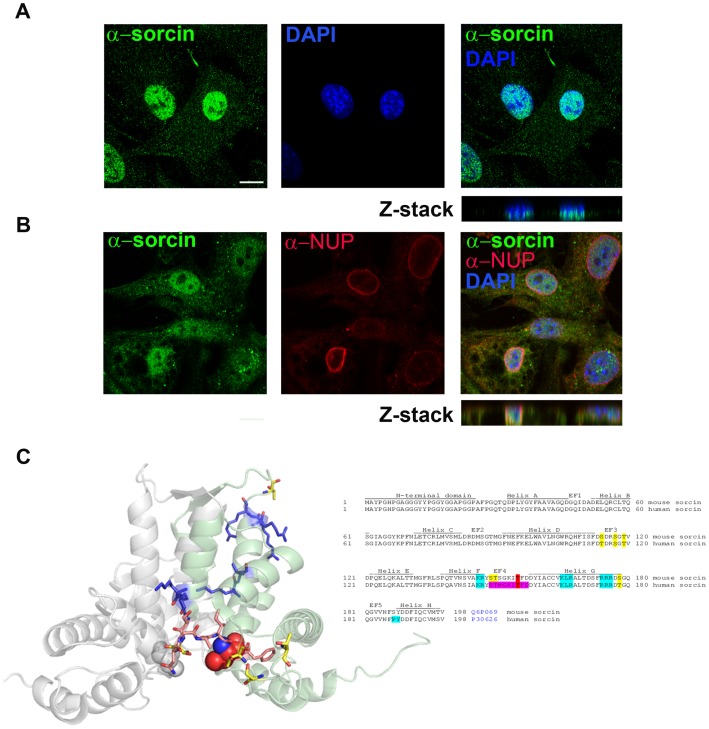
Nuclear sorcin. A. 3T3-L1 fibroblasts stained for sorcin using mouse monoclonal specific antibodies and for DNA using DAPI, studied by confocal microscopy. B. 3T3-L1 fibroblasts stained for sorcin using rabbit polyclonal specific antibodies and for the nuclear envelope and DNA using a mouse monoclonal anti-NUP antibody and DAPI, respectively, studied by confocal microscopy. Note the insertion of sorcin antibodies to the DAPI staining in the Z stacks and the exclusion of nuclear sorcin from the nucleoli. *Bars 10 µm*. C: Sequence and secondary structure of sorcin from mouse and human, and crystal structure of human sorcin (Pdb: 1JUO). The sequence is well conserved between the two species. The only differences concern a few phosphorylatable residues (serine or threonine). Mutated residues are bold, the putative Nuclear Localization Sequence residues are blue; PLK binding region is purple; T155 is red CPK in the crystal structure.

**Figure 3:**
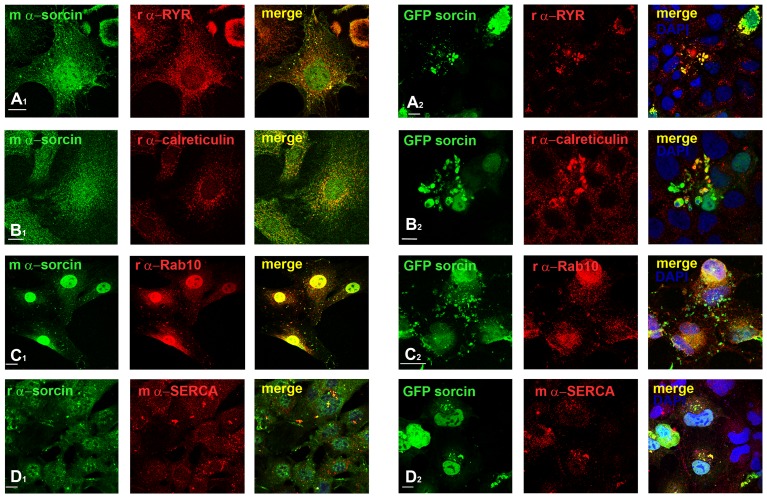
During interphase sorcin is located in the nucleus and in vesicles and macrovesicles, where it interacts with RYR, calreticulin, Rab10 and SERCA. A1: Accumulation of sorcin in RYR-positive ER and cytoplasmic vesicles. 3T3-L1 fibroblasts stained with mouse monoclonal α-sorcin and rabbit polyclonal α-RYR show colocalization of both proteins in ER and cytoplasmic vesicles and the exclusion of RYR from the sorcin-positive plasma membrane and nucleus. A2: 3T3-L1 fibroblasts transfected for 15 h with pGFPsorcin, incubated the last 3 h with 10 µg/ml cycloheximide and stained with rabbit polyclonal RYR-specific antibodies. DNA was stained using DAPI. Note the perfect colocalization of RYR in sorcin specific macrovesicles. B1: Partial colocalization of sorcin with the marker of ER calreticulin in the ER-derived vesicles, and perfect colocalization in the cytosolic microvesicles. B2: 3T3-L1 fibroblasts transfected for 12 h with pGFPsorcin as above, stained with rabbit polyclonal α-calreticulin specific antibodies. The calreticulin antibody stains specifically the cytosolic macrovesicles formed by GFPsorcin expression. C1: Sorcin and Rab10 colocalize in the nuclear envelope, ER tubules and vesicles, and macrovesicles. C2: 3T3-L1 fibroblasts transfected with pGFPsorcin as above, stained with rabbit polyclonal α-Rab10 antibodies. There is a significant Rab10 accumulation in the sites of ectopic sorcin expression, i.e. vesicles, macrovesicles and plasma membrane. D1: Sorcin and SERCA colocalize in ER tubules and vesicles, and macrovesicles. D2: 3T3-L1 fibroblasts transfected with pGFPsorcin as above, stained with mouse monoclonal α-SERCA antibodies. SERCA accumulates in the sites of ectopic sorcin expression, i.e. nuclei, vesicles, macrovesicles and plasma membrane. *Bars 10 µm*.

The 22 kda sorcin expression was confirmed by immunoprecipitation in 3T3L1 cell extract using the rabbit anti-sorcin, and the mouse monoclonal anti-sorcin for the western blotting detection (Fig. 1B). Immunofluorescence confocal microscopy studies using rabbit polyclonal and mouse monoclonal antibodies indicated that sorcin is localized in the nucleus, in the ER and cytoplasmic vesicles, and in plasma membrane (Figures 1, S2). In the nucleus sorcin was uniformly distributed in a speckled fashion and was excluded from the nucleoli (Figure 2). Transfection experiments confirmed the localization of exogenous sorcin and GFP-sorcin in the nucleus, ER and plasma membrane (Figures 1, S2). The nuclear localization of sorcin was consistent with the high number of nuclear proteins among the sorcin interactors identified in the protein array study (Table S2). In a time-course transfection experiment we observed that between 15 and 18 h of transfection, sorcin was first detected in the endoplasmic reticulum (ER) wrapping the nuclear envelope and in the nucleus, and then in vesicles, before the formation of large patches (Figures 1, 3). 11 ER proteins were found in sorcin interactome, according to the Gene NCBI database (Table S2), in addition to RYR and SERCA, already shown to interact with sorcin in sarcoplasmic or endoplasmic reticulum [11], [39].

In cell fractionation experiments sorcin was recovered with the fractions enriched in endoplasmic reticulum (ER) and low-density microsomes (LDM), and a considerable amount within the nuclear fraction (Figure 1C). The efficiency of the cell fractionation was tested by western blot analysis using specific antibodies for each compartment. The nuclear localization of sorcin was likely to reside on a stretch of positively charged residues that conform the EF4-EF5 region, involved in target recognition [40] (Figure 2C), very similar to typical nuclear localization sequences, which usually consist of one or more short sequences of positively charged lysine or arginine residues exposed on the protein surface, or a central hydrophobic or basic motif followed by a C-terminal R/H/KX_(2–5)_PY consensus sequence [41]–[43].

### Sorcin in ER and ER-derived vesicles

Sorcin was found in cytosolic vesicles rich in lipid rafts, together with Annexins 7 and 11, and to participate in the process of vesicle release from erythrocytes [44]. In 3T3-L1 fibroblasts and Huh7 cells we found that the sorcin-positive vesicles were rather large and heterogeneous in size (400 nm-3 µm diameter) (Figure S2), and their size was increased up to 6 µm upon transfection in the same cell lines with vectors expressing sorcin (Figures 1, 3). In the cardiomyocytes, sorcin interacts with sarcoplasmic reticulum calcium channels, such as RYR and SERCA, participating in their regulation [11], [12]. In agreement with these observation, we found that sorcin colocalized with RYR and SERCA in small and large vesicles and in patches [45], that contained the ER marker calreticulin (Figure 3A, 3B, 3D). Sorcin only colocalized partly with the other ER marker calnexin (Figure S3). We therefore concluded that the vesicles were ER-derived. Sorcin-containing ER vesicles and patches were shown to contain high amounts of calcium, as expected for ER-derived structures (Figure 1). Ectopic overexpression of sorcin increased not only the dimensions of the ER-derived vesicles, but also the calcium content of the ER sorcin-containing vesicles; sorcin overexpression can lead to calcium overload of ER and partial ER fragmentation (Figures 1D, 3). Furthermore, sorcin colocalized with Rab10 in the ER vesicles [46] (Figure 3C), and in the midbody (see below). Moreover, vesicular sorcin colocalized with the Rab10 and partly with Rab11, which are found in the ER and endosomes, and was excluded from the early and late endosomes and from caveolae, stained with antibodies against EEa1, Rab7 and caveolin-1, respectively (Figure S3). Ectopic overexpression of sorcin further increased the colocalization between sorcin and RYR, SERCA, calreticulin and Rab10, which accumulated in the large vesicles and patches (Figure 3). Sorcin was not found in mitochondria in the cell lines used in the work (data not shown), as has been observed for a short (18 kDa) sorcin variant in human colon carcinoma cells by Landriscina et al. [47].

### Sorcin in mitosis

**Figure 4:**
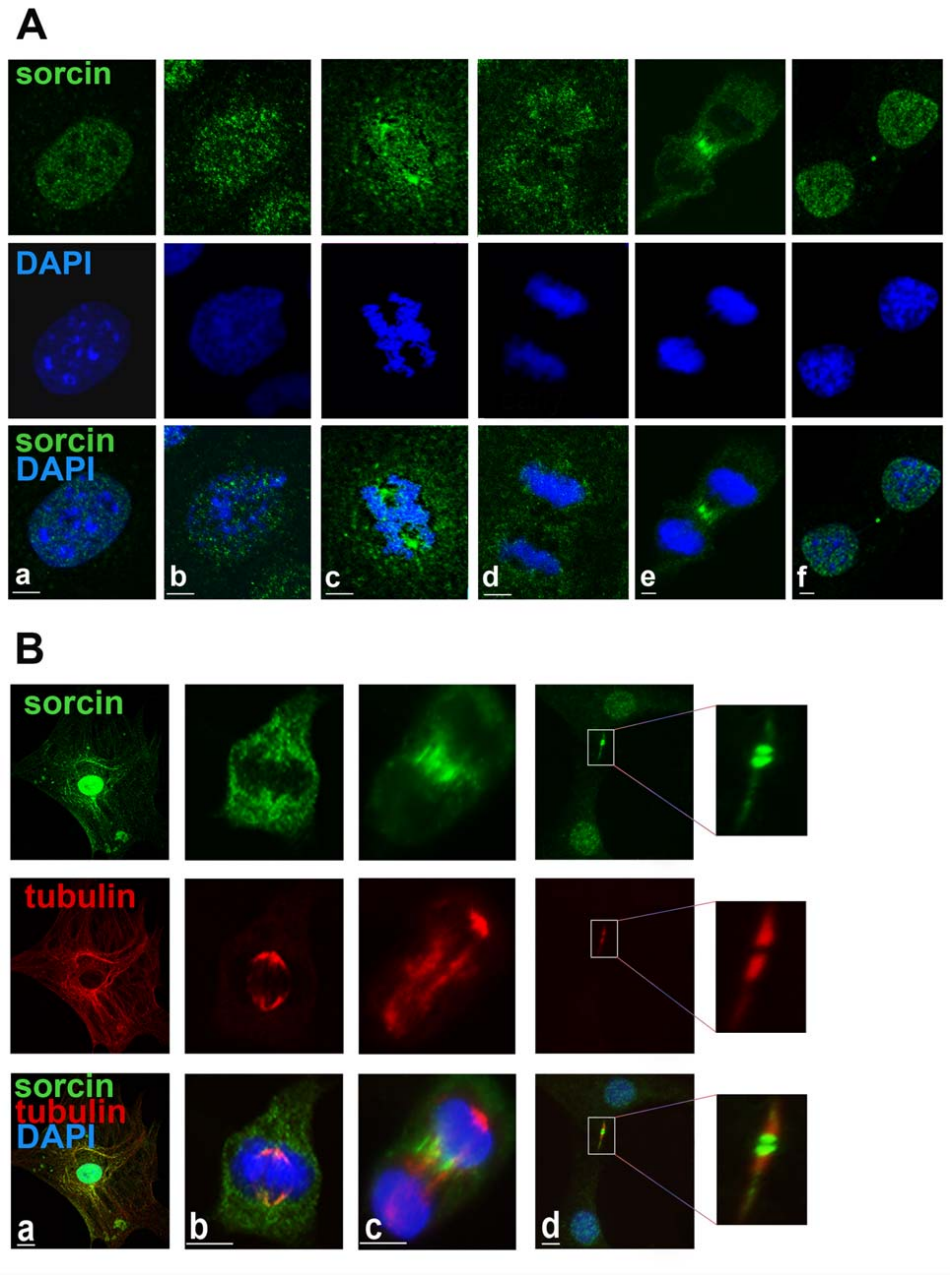
Distribution of sorcin in cell cycle. A: Interphase and mitotic 3T3-L1 fibroblasts stained for sorcin using the mouse monoclonal antibody. DNA stained using DAPI. Note the dispersion of nuclear sorcin throughout the cell after prophase, and its concentration in the cleavage furrow of cells in late telophase before its accumulation in the midbody and its reentrance in the nucleus in cells in cytokinesis. *Bars: 5 µm*. B: Sorcin association with tubulin-based structures. Mitotic mouse 3T3-L1 fibroblasts simultaneously stained for sorcin, tubulin and DNA in interphase (a), metaphase (b), telophase (c) and before abscission (d). Note the association of sorcin with microtubular structures during interphase, with the poles of the mitotic spindle during metaphase, with the cleavage furrow of cells in telophase, its accumulation in the midbody and reentrance in the nucleus of cells in cytokinesis, before completion of mitosis. Observe the flanking of sorcin by tubulin. *Bars: 10 µm*.

**Figure 5:**
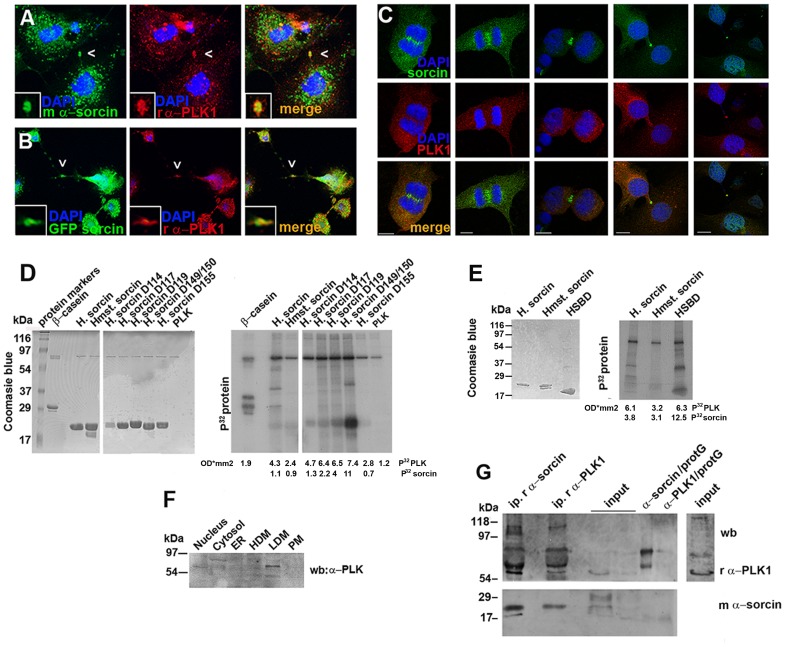
Localization of sorcin and PLK1 within the midbody and in vitro PLK1 kinase assay. A: Cells in cytokinesis were stained for sorcin and PLK1. Observe sorcin accumulation on both sides of PLK1 (white arrows). B: Cos7 cells expressing GFP-sorcin were stained for PLK1. *Bars 10 µm*. C: PLK1 localizations in the midbody in 3T3-L1 fibroblasts before abscission. Sorcin is localized in the region flanking the bulge of the midbody. PLK1 localization is more variable, but it is often found in the bulge region of the midbody. D, E: The products of the *in vitro* kinase assay were analysed using SDS/PAGE and colored with Coomassie Blue (left), following by autoradiography (right). PLK substrates used are 200 ng/μL: dephospho-casein, human sorcin (HS), hamster sorcin (Hst), human sorcin mutants D114, D117, D119, D149150, D155 and sorcin C-terminal calcium binding domain (HSBD). PLK1 kinase activity was measured in a radiometric assay according to manufacturer's instructions. The P^32^-labeled PLK and sorcin protein bands were quantified and the values added below each lane. F: Analysis of the cellular fractions nuclei, cytosol, ER, HDM, LDM and plasma membranes by SDS-PAGE and western blot using rabbit antibodies against PLK1. **G:** Western blot analysis of immunoprecipitated proteins from 3T3L1 cell lysate (500 μg) using the rabbit polyclonal α-sorcin (i.p. r α-sorcin) and the rabbit polyclonal α-PLK (i.p. r α-PLK), of input fraction 20 μg and 10 μg (input), of rabbit polyclonal α-sorcin bound to protein G (α-sorcin/protG) and rabbit polyclonal α-PLK bound to protein G (α-PLK/protG) and the input fraction chemiluminescent detected 10 times more than the above input.

In the interphase, a large fraction of sorcin appears to be nuclear, with the cytosolic vesicular sorcin fraction being associated with microtubules (Figure 4B). During the cell cycle, sorcin exhibits a characteristic behavior (Figures 4, 5). In prophase and immediately after disruption of the nuclear envelope, sorcin was not associated with the DNA, but it was accumulated in the apical region of mitotic spindle (Figure 4Ab, 4Bb). In metaphase, most sorcin was found associated with the spindle and, upon chromosome separation, a large fraction of sorcin accumulated in the central zone of the spindle (Figure 4). Subsequently, in the early telophase sorcin was found in the cleavage furrow (Figs. 4Ae, 4Bc). In late telophase, most sorcin was relocalized to the reforming nuclei, but a significant fraction was found flanking the central part of the midbody (Figures 4Af, 4Bd, 5, S4). The midbody proteins that interact with sorcin are listed in Table S3. Double staining microscopy experiments revealed the partial colocalization of sorcin with PLK1 and Rab11 (Figs. 5, S4) in the ER cytosolic vesicles, especially around the nucleus, and in the midbody. In addition sorcin colocalized with Rab10 in both ER vesicular structures and in the midbody (Figure 3, S4). These are the first data demonstrating the presence of Rab10 in the midbody. Rab10 has been published to be associated with primary cilia and the exocyst complex that is required for secretory vesicle-mediated abscission [48], [49].

These data show the distribution of ER-derived vesicles during mammalian cell division and provide evidence of the localization of ER proteins in the cleavage furrow and in the midbody.

### Sorcin interacts with PLK1, is phosphorylated by PLK1 and controls PLK1 autophosphorylation

Sorcin colocalizes with PLK1 in the midbody, and expression of GFP-sorcin in Cos7 cells confirmed the match between sorcin and PLK1 (Figure 5B). The cell doublets and the unusual length of the midbody in the cells transfected with GFP-sorcin (Figure 5B) indicate that overexpressed sorcin may interfere with cell abscission.

Furthermore, *in vitro* array-based experiments showed that sorcin interacts physically with PLK1. The sorcin-PLK1 interaction was calcium-dependent, as showed by the 1.5-fold increase in the interaction in the presence of 1 mM calcium, as compared to the interaction in the presence of EDTA (Table S2). To validate the *in vitro* array results we carried out cell fractionation experiments, identifying PLK in the nucleus and in the low density microsomes, two compartments rich in sorcin (Figures 1C and 5F) and immunoprecipitation experiments, detecting sorcin in the immunoprecipitation products of the rabbit α-PLK1 and PLK1 in the immunoprecipitation products of the rabbit α-sorcin (Figure 5G).

*In vitro* phosphorylation studies demonstrated that sorcin is phosphorylated by PLK1 (Figure 5D). The consensus sequence of the PLK1 phosphorylation motif D/E-X-pS/pT-Φ-X-D/E (X, any amino acid; Φ, a hydrophobic amino acid) has two acidic amino acids at positions −2 and +3 of the phosphorylated Ser/Thr, preferred but not essential for kinase recognition, and one hydrophobic amino acid at position +1 [50]. Using site directed mutagenesis we substituted Asp for Ser/Thr residues, to mimic phosphorylation. *In vitro* phosphorylation studies showed that the substitution T155D decreases sorcin phosphorylation by PLK1 (Figure 5D), therefore indicating that T155 was phosphorylated by PLK1. Thr155 is in the 149–158 STSGKITFDD stretch, which is part of the sequence that comes into contact with the N-terminal sorcin domain or with sorcin molecular targets [40]. In addition, the sequence contains the S-[pS/pT]-P/X motif that mediates the interaction between the PLK1 substrates and the PLK1-box domains [51] (Figure 2).

Mutation of Ser149 and Thr150 in aspartic residues, which mimics their phosphorylation, increased substantially the phosphorylation of sorcin by PLK1, showing that phosphorylation of these residues by other kinases increases the number of negative charges around T155, and may activate sorcin phosphorylation by PLK1 (Figure 5D). *In vitro* phosphorylation of the C-terminal sorcin calcium-binding domain (HSCBD) was increased by about 4 times as compared to sorcin phosphorylation (Figure 5E), showing that the N-terminal domain decreases the accessibility of the phosphorylation site, as already demonstrated for Protein Kinase A [40].

Furthermore, the interaction with sorcin stimulates PLK1 activity, as indicated by the large increase in PLK1 autophosphorylation (Figure 5D), which was about 2–4 times higher than the autophosphorylation observed in the presence of casein, a known target of PLK1 which is able to activate PLK1 autophosphorylation [52].

### Sorcin silencing

**Figure 6:**
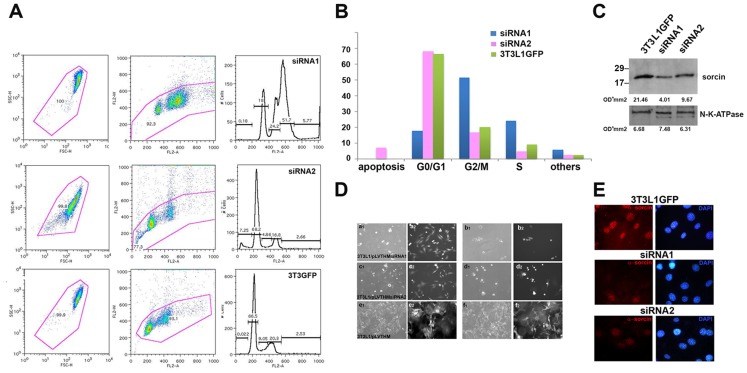
Silencing of sorcin results in cell arrest and cell death. A: sorcin siRNAs 1,2 and control 3T3-L1 fibroblasts (3T3-L1GFP) analysis by cytometry. B: Percentage of sorcin siRNAs 1,2 and 3T3-L1GFP cells in apoptosis, G0/G1, G2/M and S phase of the cell cycle; C: Expression levels of sorcin in 3T3-L1 fibroblasts infected with sorcin siRNA1 (86±4% silencing) and siRNA2 (64±11% silencing) and with lentivirus control (3T3-L1GFP) vector. The gene silencing efficacy of siRNAs was measured by three different densitometry quantifications, and using Na-ATPase as a reference. A representative measure is shown. D. Phase contrast (a1-f1) and GFP fluorescence images (a2-f2) upon silencing. Images of 3T3-L1 fibroblasts infected with sorcin siRNAs 1 (a, b) siRNAs 2 (c, d) and control 3T3-L1 fibroblasts after infection with empty pLVTHM (e, f). a, c and e show cells after a single infection; b, d and f show the fibroblasts after two cycles of infection.E: Fluorescence of control 3T3-L1 fibroblasts and fibroblasts infected with sorcin siRNAs 1 and 2, stained for sorcin. *Bars: 10 µm*.

Two different siRNAs constructs were used (siRNA1, siRNA2) to silence the mRNA expression of sorcin in 3T3-L1 fibroblasts (Figure 6). The gene silencing efficacy of siRNA1 was stronger (86±4%) than that of siRNA2 (64±11%). Downregulation of sorcin expression resulted in a high amount of cell death.

The sorcin silencing achieved using siRNA1 was sufficient to elicit profound effects on cell growth, mitosis and cytokinesis. This result was supported by flow cytometry (Figure 6A, 6B). The analysis was made only on the cells remained attached to the plate, excluding the floating cells; therefore the number of apoptotic cells is underestimated in the analysis. Knockdown of sorcin in 3T3-L1 fibroblasts above 75% resulted in an increase in the G2/M-phase fraction, indicating a partial blockage in the entrance in mitosis. Changes in the shape of the cells, with the presence of multiple aggregates, and with evident defects in the abscission processes were also observed upon sorcin silencing (Figure 6D). Two cycles of infection invariantly cause cell death (Figure 6D). Sorcin silencing with siRNA1 has important structural consequences on 3T3-L1 fibroblasts mitotic processes and hampers abscission: we could find no telophase, nor midbodies, in cells expressing very low levels of sorcin upon silencing with siRNA1. The FACS analysis of the cells treated with the siRNA2 show normal cell cycle phase but a significant number of apoptotic cells (Figure 6A).

## Discussion

This work is the first study on the role of sorcin in the cell cycle. The presence of transient and localized increases in Ca^2+^ concentration in mitosis has been demonstrated for diverse cell types [2], [3], while a slower rise of the ion concentration, with a peak before abscission, has been demonstrated to contribute to mitosis regulation in many mammalian cells [53]. In 3T3-L1 fibroblasts, nuclear envelope breakdown is linked to a Ca^2+^ increase and anaphase is a calcium-modulated event, not triggered by brief calcium transients, but accompanied by a sustained elevation of Ca^2+^ concentration, which depends on the presence of an intact spindle [4], [54].

Above all, calcium regulates the activity of many kinases which participate in mitosis progression and regulation. Sorcin both interacts in a calcium-dependent fashion with a series of targets important for cytokinesis, and participates in the phosphorylation-dephosphorylation network that regulates mitosis and cytokinesis. We have shown that in fibroblasts sorcin is located primarily in the nucleus but is found also in cytosolic ER vesicles and macrovesicles, and is able to establish interactions with many protein targets. These targets include protein kinases localized in the spindle and in the midbody, and involved in cell cycle regulation, such as PLK1, Aurora A and Aurora B. Sorcin contains potential phosphorylation sites for PLK1, Aurora A and Aurora B, which are among sorcin interactors *in vitro*. We have demonstrated that sorcin can be phosphorylated by PLK1 and is able to induce PLK1 autophosphorylation, which contributes to kinase regulation. PLK1 plays multiple functions during mitosis, has a dynamic localization in midbody and midzone and is degraded before cytokinesis. Inhibition of its kinase activity blocks midbody protein relocalizations and stem body formation, culminating in breakdown of the midbody and in­vasion of the intracellular bridge by dynamic microtubules [55]. PLK1 is activated *via* Aurora A-dependent Thr210 phosphorylation [56], which can be initially detected on centrosomes in G2; it peaks in prometaphase and gradually disappears from centrosomes during anaphase. Dephosphorylation of phospho-Thr210 at centrosomes is probably mediated by protein phosphatase 1C (PP1C) [57], a sorcin interactor (PPP1R14A and PPP1R8, Table S1). Once activated, PLK1 is stimulated by binding target proteins. Sorcin interacts with PLK1 both *in vitro* and *in vivo*, and this interaction is likely to have significant consequences in mitosis, as indicated by the profound effects of sorcin silencing, which are similar to those of PLK1 silencing [58], i.e. accumulation of rounded-up mitotic cells, increase in the amount of G2-M cells and decrease in cell growth. Taken together, these data indicate that sorcin and PLK1 may participate in the same regulative processes, and that sorcin indeed regulates PLK1.

Many of the proteins interacting with sorcin and located in the midbody, listed in Table S3, bind calcium or other divalent cations, or are calcium-activated, such as Aurora A, which plays a role in late mitotic events as completion of cytokinesis [59]. Release of Ca^2+^ from ER is known to rapidly and transiently activate Aurora A by direct Ca^2+^-dependent calmodulin binding [60]. In sorcin, both Ser149 and Ser178 are predicted to be possible targets for Aurora A-dependent phosphorylation [61]. Sorcin interacts *in vitro* with Aurora B, and Ser149 is part of a consensus sequence of Aurora B-dependent phosphorylation. Abscission occurs at the outer edge of the Aurora B-binding region [55], and sorcin appears to concentrate and to localize in such regions of the midbody, during cytokinesis (Figures 4–5).

A similar dynamic nuclear and midbody localization has been found for calcium/calmodulin kinase II (CaMKII), which has been demonstrated to interact with sorcin and to phosphorylate sorcin in the presence of Ca^2+^. In addition, dephosphorylated sorcin significantly inhibits CaMKII activity [62], [63]. Only a very small fraction of protein interactors is shared by sorcin and calmodulin [64]. Sorcin participates in Ca^2+^-dependent kinases regulation, and links phosphorylation-dependent regulation to Ca^2+^-dependent processes, in a different fashion with respect to calmodulin, though being based on similar mechanisms.

Sorcin is able to interact with and to dynamically colocalize with calcium-activated proteins as RyR, Rab10 (identified for the first time as a component of the midbody) and Rab11, which play a role in vesicle trafficking and in the regulation of mitosis and cytokinesis. The accumulation of calcium-activated proteins and of sorcin-containing calcium stores at the cleavage furrow and subsequently at the midbody, a sub-membrane highly dynamic structure, indicates that local calcium concentration is presumably rather high, that membrane addition during cytokinesis and abscission are calcium-dependent processes, and that sorcin participates in the regulation of such processes.

**Figure 7:**
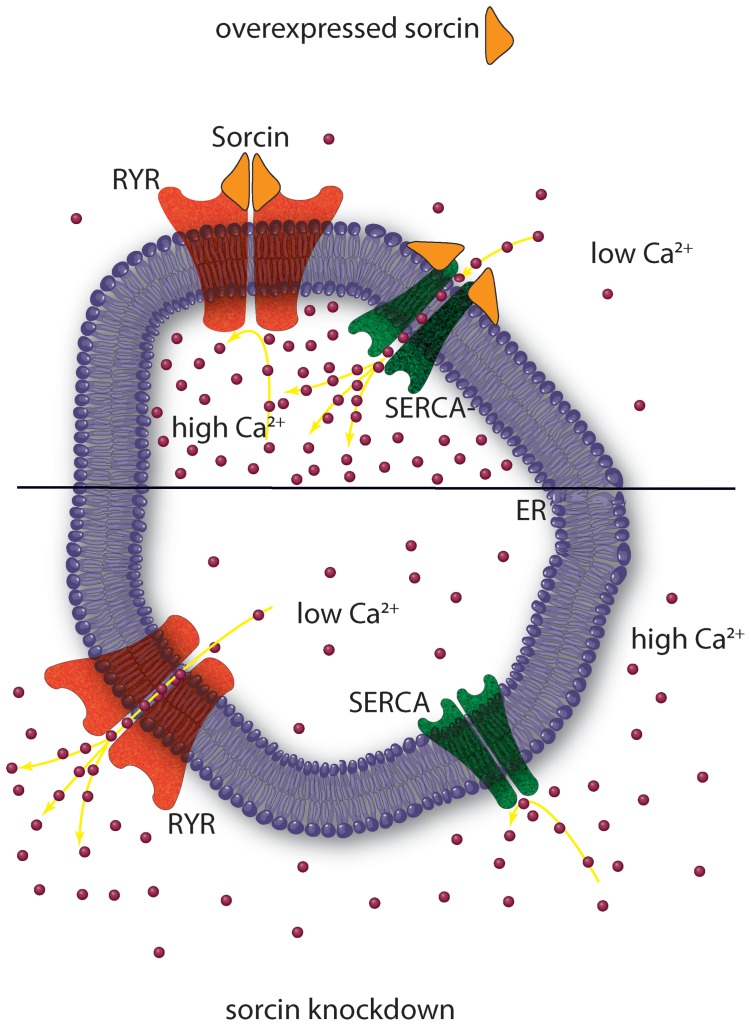
Sorcin inhibits the release of Ca^2+^ from ER and increases calcium load of the store by inhibiting RYR and activating SERCA. Sorcin overexpression (top) alters calcium homeostasis in the cell, increasing the ion content of the ER, and decreasing cytosolic free Ca^2+^ concentration. Sorcin knockdown (bottom) has opposite effects.

The heterogeneity of the cytoplasmic sorcin vesicles, the association of Rab10 and Rab11 to the GLUT4 compartments [65], and the partial colocalization with Rab11, one of the proteins of sorcin interactome (Figure S3), will be investigated to study whether a part of sorcin vesicles belongs to the recycling endosomes and/or to secretory granules [66], [67]. Further studies are also required to elucidate the composition and the destination of sorcin vesicles, as caveolin-1, found in the secreted microvesicles in 3T3-L1 adipocytes [68], is not a component of the ectopically expressed sorcin-containing vesicles in 3T3-L1 fibroblasts. Sorcin vesicular structures, however, are mostly ER or ER-derived, since they also contain Rab10, RYR, SERCA and calreticulin, and are rich in calcium, as expected for ER (Figure 2). In order to establish interactions with proteins at the ER or at plasma membrane (sites where local calcium concentration is high), sorcin binds calcium. Ca^2+^-bound sorcin negatively regulates the release of Ca^2+^ from ER and increases calcium load of the store by inhibiting RYR and activating SERCA [10]–[12]. Sorcin alter calcium homeostasis in the cell, increasing the ion content of the calcium stores, and decreasing cytosolic free Ca^2+^ concentration (Figure 7) [69]. Sorcin increases the accumulation of Ca^2+^ in the ER, preventing ER stress, and is upregulated under conditions of ER stress [47]. Ectopic overexpression of sorcin (Figures 3, 4) increases the dimensions of the ER-derived vesicles, determines increase in calcium load of ER and may even lead to partial ER fragmentation upon calcium overload. The presence of sorcin-containing calcium-rich ER vesicles at the cleavage furrow and at the midbody strongly links calcium signaling with vesicular trafficking.

We have also demonstrated that sorcin is essential for mitotic progression and cytokinesis, and that sorcin silencing results in increase in the number of rounded polynucleated cells, and cell death. However, different levels of silencing determine diverse effects on cell cycle: a 85–90% sorcin silencing, induced by siRNA1, results in a strong decrease in sorcin in all compartments (nucleus included) (Fig. 6E), and determines strong mitotic effects, major defects in cytokinesis and blockage of cell progression in G2/M, while the siRNA2 silencing result in a milder, non mitotic phenotype, with an increase of apoptosis and cell death, possibly due to a decrease in the sorcin cytosolic fraction (rather than in the sorcin nuclear fraction), and to the consequent effects on ER stress. A possible differential effect on the two different splicing sorcin variant described in literature cannot be excluded, since a 18 kDa mitochondrial sorcin form has been demonstrated to interact with Trap1 and to protect cells against apoptosis [47].

Sorcin plays important roles in Ca^2+^-loaded vesicle trafficking and mitosis, regulates calcium homeostasis, regulates PLK1, and can be considered an activator of cytokinesis. Cell death upon decrease in sorcin expression dovetails with the high levels of sorcin expression associated with multiple myeloma, lymphoma, breast cancer and other cancers, and with sorcin upregulation in Multi Drug Resistant (MDR) tumor cells which are resistant to apoptosis onset. High levels of sorcin may serve to prevent ER stress, to activate mitosis and cytokinesis processes, and to help development of resistance towards chemotherapeutics in cancer cells [18]–[26], [47], [70], [71]. Thus, this study indicates sorcin as a potential molecular target to sensitize resistant tumor cells.

## Supporting Information

Figure S1 **Protoarray experiments carried out with sorcin.** 10 µM A2C-sorcin-AlexaFluor 532 maleimide incubated with the array in the presence of 1 mM CaCl_2_ and in the presence of 1 mM EDTA (not shown).
(TIF)

Figure S2 **Distribution of sorcin in different cell type.** Huh7 (A1, A2) and 3T3-L1 fibroblasts (B) cells were stained with mouse monoclonal (m), rabbit polyclonal (r) specific sorcin antibodies and DAPI. Macrovesicles are indicated with white arrows. Note the comparable staining of the nucleus, cytoplasmic vesicles and plasma membrane with both antibodies. Huh7 cells (A3) transfected with pCDNA3.1 sorcin for 12 h incubated the last 3 h with 10 µg/ml cycloheximide before their staining with mouse monoclonal and rabbit polyclonal sorcin specific antibodies. DNA was stained using DAPI. A massive accumulation of the ectopically expressed sorcin is shown in the nucleus, ER, cytoplasmic vesicles and plasma membrane. *Bars: 10 µm*. C: Staining of mouse 3T3-L1 adipocytes, rat NRK fibroblasts and human 293FT embryonal kidney using specific mouse monoclonal sorcin antibody. Note the staining of the nucleus, cytosolic vesicles and plasma membrane. *Bars: 10 µm*.
(TIF)

Figure S3 **Sorcin in the vesicles.** A. Staining of sorcin with calnexin and EAA1,Rab11, Rab8 and Rab7. Note the partial co-localization between sorcin and calnexin. Rab11 partially colocalizes with sorcin in macrovesicles while EAA1 and Caveolin 1 do not colocalize with sorcin. *Bars 10 µm*.
(TIF)

Figure S4 **Rab proteins partially colocalize with sorcin in the midbody.** Rab10 and Rab11 GTPases are found in 3T3-L1 fibroblasts midbody, before abscission. Sorcin partially colocalizes with these proteins in the region flanking the bulge of the midbody.
(TIF)

Table S1 **Sorcin interactome based on ProtoArray experiments.**
(DOCX)

Table S2 **Sorcin interactome: enriched Gene Ontology categories according to BioProfiling.de.**
(DOCX)

Table S3 **Proteins interacting with sorcin and identified in the midbody.**
(DOCX)
