# Supplementary figures and images for: Sorcin Links Calcium Signaling to Vesicle Trafficking, Regulates Polo-Like Kinase 1 and Is Necessary for Mitosis
Source: PLoS One. 2014 Jan 10;9(1):e85438. doi: 10.1371/journal.pone.0085438 (PMC3888430; doi:10.1371/journal.pone.0085438)

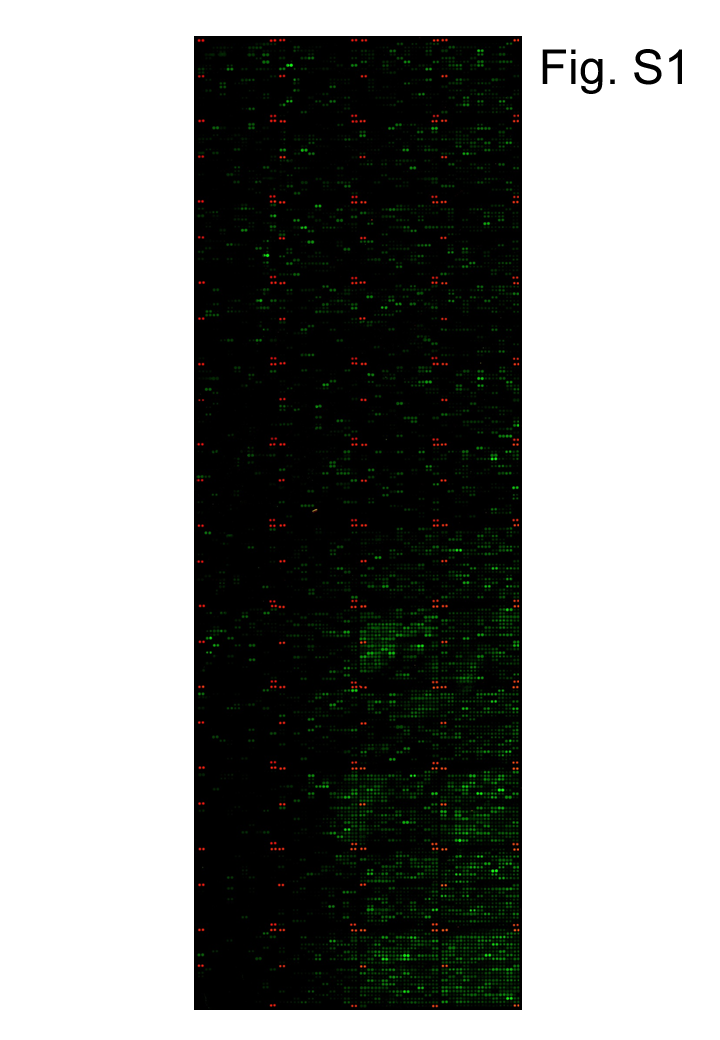

Supplement: Figure S1 — Protoarray experiments carried out with sorcin. 10 µM A2C-sorcin-AlexaFluor 532 maleimide incubated with the array in the presence of 1 mM CaCl2 and in the presence of 1 mM EDTA (not shown). (TIF) [file pone.0085438.s001.tif]

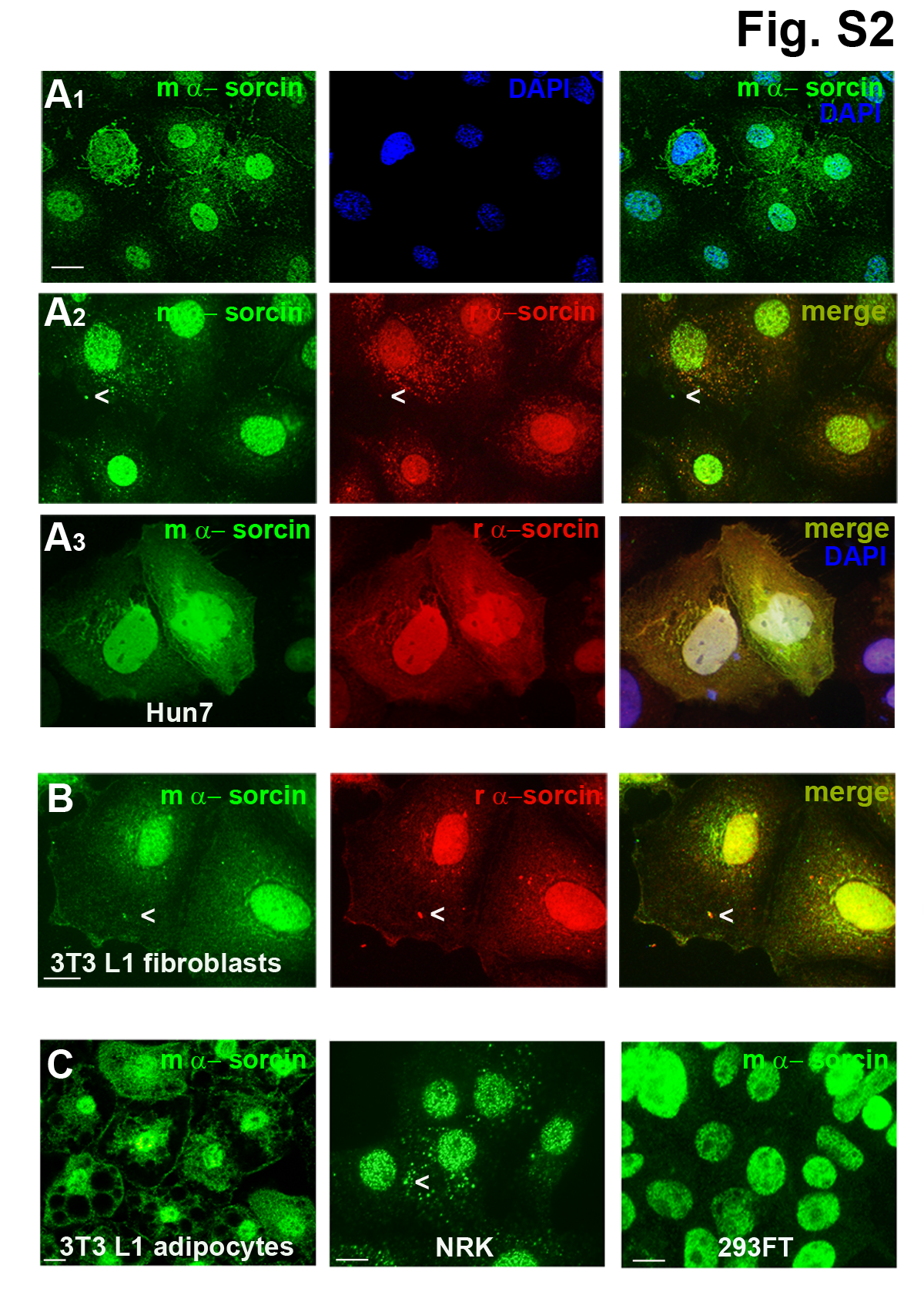

Supplement: Figure S2 — Distribution of sorcin in different cell type. Huh7 (A1, A2) and 3T3-L1 fibroblasts (B) cells were stained with mouse monoclonal (m), rabbit polyclonal (r) specific sorcin antibodies and DAPI. Macrovesicles are indicated with white arrows. Note the comparable staining of the nucleus, cytoplasmic vesicles and plasma membrane with both antibodies. Huh7 cells (A3) transfected with pCDNA3.1 sorcin for 12 h incubated the last 3 h with 10 µg/ml cycloheximide before their staining with mouse monoclonal and rabbit polyclonal sorcin specific antibodies. DNA was stained using DAPI. A massive accumulation of the ectopically expressed sorcin is shown in the nucleus, ER, cytoplasmic vesicles and plasma membrane. Bars: 10 µm. C: Staining of mouse 3T3-L1 adipocytes, rat NRK fibroblasts and human 293FT embryonal kidney using specific mouse monoclonal sorcin antibody. Note the staining of the nucleus, cytosolic vesicles and plasma membrane. Bars: 10 µm. (TIF) [file pone.0085438.s002.tif]

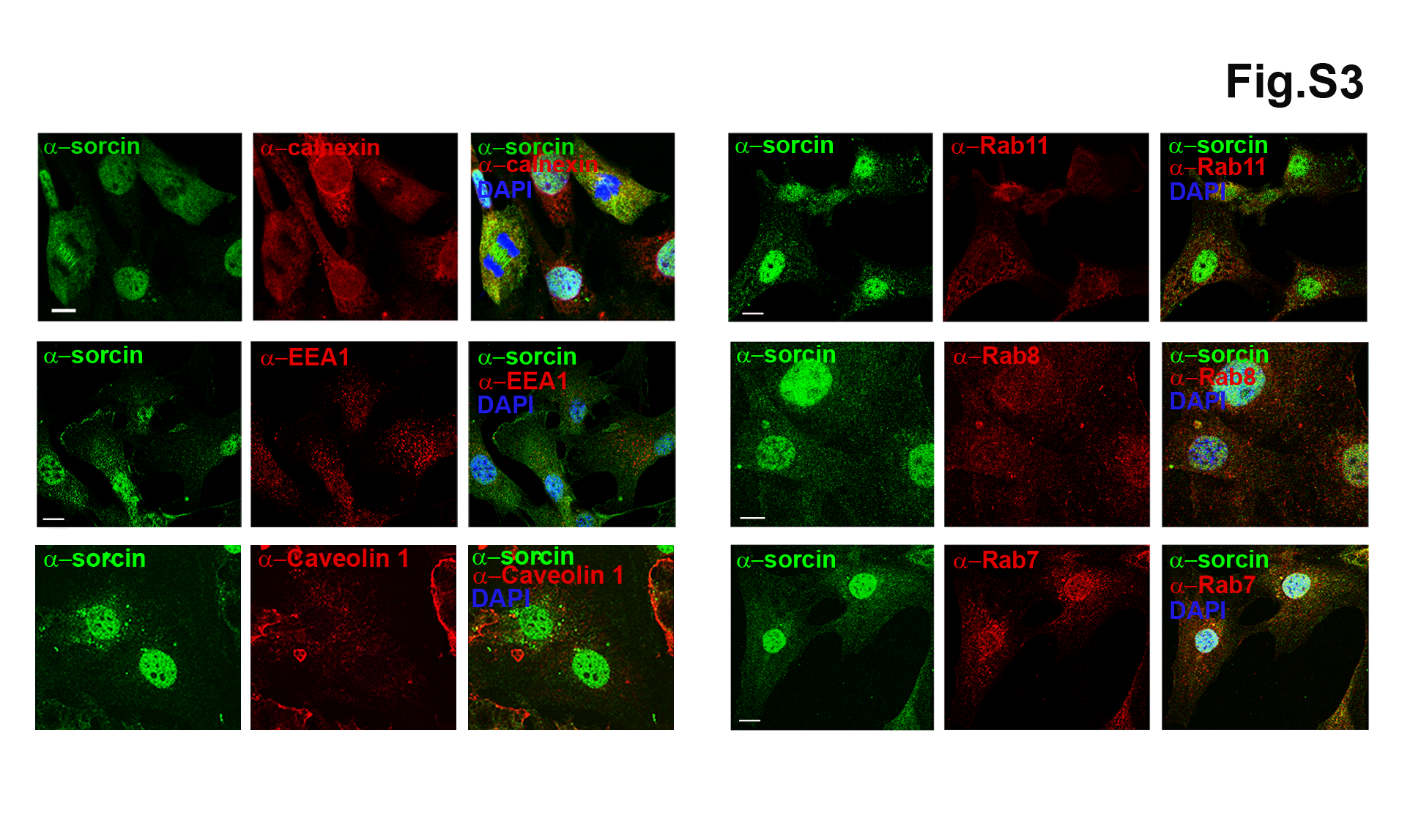

Supplement: Figure S3 — Sorcin in the vesicles. A. Staining of sorcin with calnexin and EAA1,Rab11, Rab8 and Rab7. Note the partial co-localization between sorcin and calnexin. Rab11 partially colocalizes with sorcin in macrovesicles while EAA1 and Caveolin 1 do not colocalize with sorcin. Bars 10 µm. (TIF) [file pone.0085438.s003.tif]

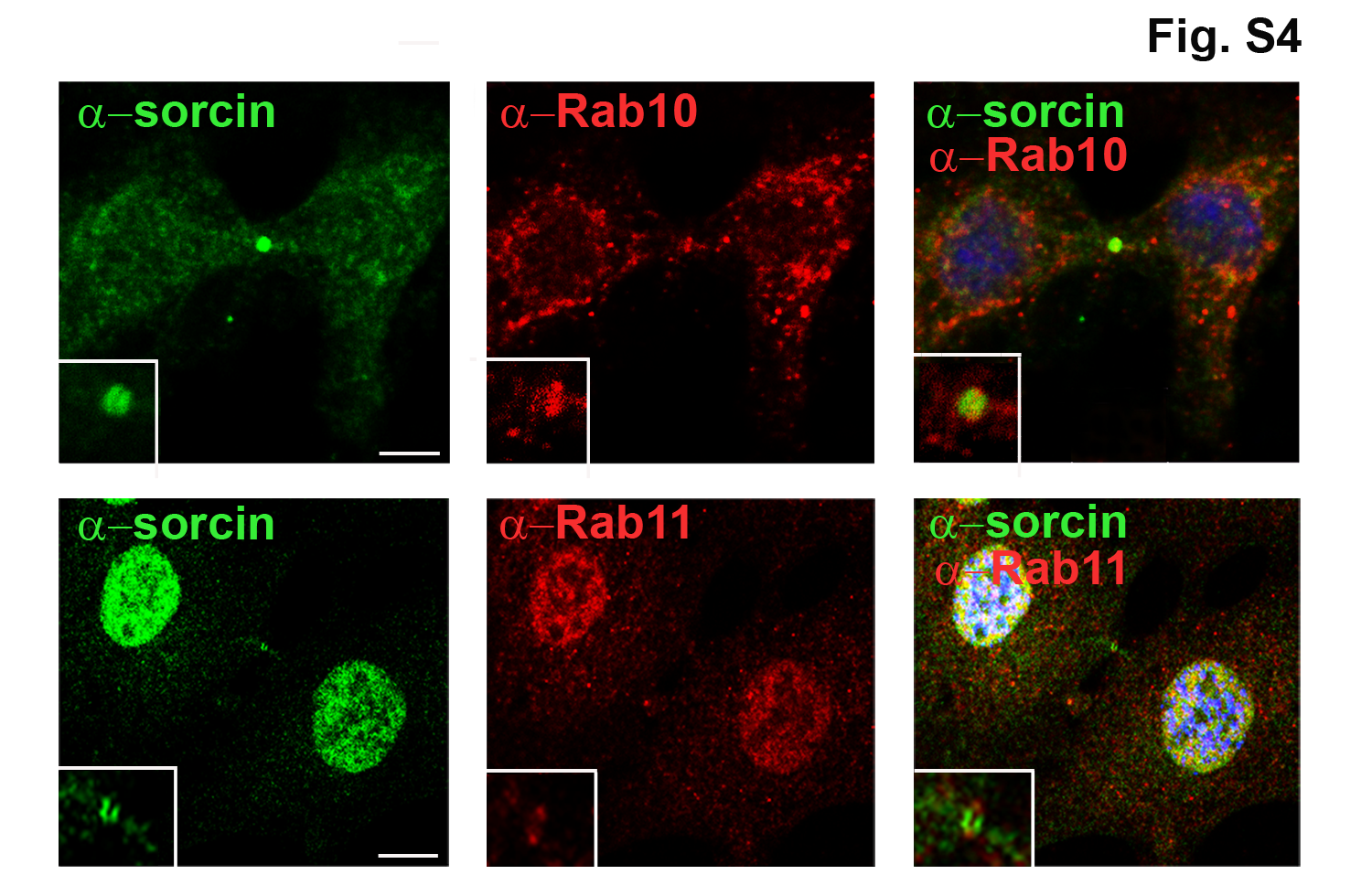

Supplement: Figure S4 — Rab proteins partially colocalize with sorcin in the midbody. Rab10 and Rab11 GTPases are found in 3T3-L1 fibroblasts midbody, before abscission. Sorcin partially colocalizes with these proteins in the region flanking the bulge of the midbody. (TIF) [file pone.0085438.s004.tif]
