# Supplementary material for: Sorcin Links Calcium Signaling to Vesicle Trafficking, Regulates Polo-Like Kinase 1 and Is Necessary for Mitosis
Source: PLoS One. 2014 Jan 10;9(1):e85438. doi: 10.1371/journal.pone.0085438 (PMC3888430; doi:10.1371/journal.pone.0085438)
Supplement: Table S1 — Sorcin interactome based on ProtoArray experiments. (DOCX) [file pone.0085438.s005.docx]

**Table S1**

**Sorcin interactome based on ProtoArray experiments.** The fluorescence signal of sorcin conjugated with Alexa532 was measured in two different experiments, carried out in the presence of 1 mM CaCl_2_ (experiment 1) and in the presence of 1 mM EDTA (experiment 2). Most known sorcin interactors are large membrane proteins, which are not included in the array.

| **Gene** | **Database ID** | **Signal 1 (+Ca^2+^)** | **Signal 2 (+EDTA)** | **Signal 1**  **/Signal 2** |
| --- | --- | --- | --- | --- |
|  |  |  |  |  |
| AAMDC | BC016854.1 | 337 | 137 | 2.46 |
| ABCF1 | BC034488.2 | 440.5 | 167.5 | 2.63 |
| ADAP2 | BC033758.1 | 272 | 220.5 | 1.23 |
| AFF4 | BC025700.1 | 528.5 | 286 | 1.85 |
| AGFG1 | BC030592.2 | 273.5 | 265 | 1.03 |
| AHDC1 | BC014394.1 | 663 | 446.5 | 1.48 |
| AHI1 | BC094800.1 | 465.5 | 198.5 | 2.35 |
| AKT2 | NM_001626.2 | 326 | 164.5 | 1.98 |
| ALDH4A1 | BC007581.1 | 392.5 | 208.5 | 1.88 |
| ANKMY2 | NM_020319.1 | 518 | 183.5 | 2.82 |
| ANXA11 | NM_001157.2 | 443 | 211.5 | 2.09 |
| APEX1 | NM_001641.2 | 259.5 | 194 | 1.34 |
| APEX2 | NM_014481.2 | 349 | 258 | 1.35 |
| ASXL1 | BC064984.1 | 511.5 | 345.5 | 1.48 |
| ATF6 | BC014969.1 | 407.5 | 153 | 2.66 |
| AURKA | NM_003600.1 | 417.5 | 213 | 1.96 |
| AURKB | BC000442.1 | 232.5 | 180.5 | 1.29 |
| BAIAP2 | BC014020.1 | 452.5 | 162 | 2.79 |
| BMX | BC016652.1 | 384 | 155 | 2.48 |
| C11orf52 | NM_080659.1 | 344.5 | 219 | 1.57 |
| C11orf63 | BC009820.2 | 528 | 320.5 | 1.65 |
| C11orf63 | NM_199124.1 | 1413.5 | 652.5 | 2.17 |
| C17orf85 | NM_018553.1 | 498 | 278.5 | 1.79 |
| C19orf43 | NM_024038.2 | 849 | 517.5 | 1.64 |
| C1orf116 | NM_023938.4 | 620 | 224.5 | 2.76 |
| C20orf198 | NM_139016.2 | 334 | 216 | 1.55 |
| C5orf55 | BC029796.1 | 401.5 | 227 | 1.77 |
| C8orf59 | BC032347.1 | 544 | 223.5 | 2.43 |
| CACNB1 | NM_000723.3 | 330 | 170.5 | 1.94 |
| CALU | NM_001219.2 | 330 | 227 | 1.45 |
| CAPRIN1 | NM_005898.4 | 350 | 146.5 | 2.39 |
| CASK | NM_003688.1 | 273.5 | 182 | 1.50 |
| CCDC23 | BC029427.1 | 359 | 181.5 | 1.98 |
| CDC7 | NM_003503.2 | 295 | 183 | 1.61 |
| CDK7 | BC005298.1 | 341.5 | 149.5 | 2.28 |
| CFDP1 | NM_006324.1 | 414.5 | 159.5 | 2.60 |
| CHGB | NM_001819.1 | 303 | 191.5 | 1.58 |
| CMAS | BC016609.1 | 338 | 138.5 | 2.44 |
| COX6A2 | NM_005205.2 | 206.5 | 189 | 1.09 |
| CPNE1 | NM_003915.2 | 390.5 | 105.5 | 3.70 |
| CRIP2 | NM_001312.2 | 271.5 | 218 | 1.25 |
| CSAG1 | BC059947.1 | 446 | 285 | 1.56 |
| CSNK2A1 | NM_001895.1 | 371.5 | 194.5 | 1.91 |
| CSNK2A2 | NM_001896.2 | 322.5 | 191 | 1.69 |
| CSPP1 | NM_024790.2 | 391 | 143 | 2.73 |
| CSRP1 | NM_004078.1 | 336.5 | 207 | 1.63 |
| CTNND1 | BC010501.1 | 540 | 346.5 | 1.56 |
| CTTN | NM_138565.1 | 773 | 1467 | 0.53 |
| CWC15 | BC040946.1 | 340 | 177.5 | 1.92 |
| DFFB | NM_001004285.1 | 362.5 | 139.5 | 2.60 |
| DNAJC8 | NM_014280.1 | 380.5 | 256.5 | 1.48 |
| DYNC2LI1 | BC006969.1 | 245 | 180.5 | 1.36 |
| EHHADH | NM_001966.2 | 603 | 303.5 | 1.99 |
| EIF1AX | NM_001412.2 | 603 | 437.5 | 1.38 |
| EIF1AY | BC005248.1 | 433 | 219.5 | 1.97 |
| EIF2A | BC011885.1 | 376.5 | 164 | 2.30 |
| ELOF1 | NM_032377.2 | 361 | 260 | 1.39 |
| ENKUR | NM_145010.1 | 356 | 178.5 | 1.99 |
| EPB41L4A | NM_022140.2 | 514 | 329.5 | 1.56 |
| ERI1 | NM_153332.2 | 378.5 | 167.5 | 2.26 |
| ETV5 | NM_004454.1 | 411.5 | 171 | 2.41 |
| FAM107A | NM_007177.1 | 788 | 231.5 | 3.40 |
| FAM213A | BC005871.2 | 368.5 | 204.5 | 1.80 |
| FAM76B | NM_144664.3 | 327 | 230 | 1.42 |
| FERMT3 | BC013366.2 | 337 | 153.5 | 2.20 |
| FGF12 | NM_021032.2 | 350 | 211 | 1.66 |
| FGF13 | NM_033642.1 | 417 | 155 | 2.69 |
| FHL1 | NM_001449.2 | 372.5 | 167.5 | 2.22 |
| FKBP3 | NM_002013.2 | 331.5 | 218.5 | 1.52 |
| FRG1B | NM_207350.1 | 375.5 | 198 | 1.90 |
| GABARAP | NM_007278.1 | 377.5 | 240 | 1.57 |
| GABARAPL1 | NM_031412.1 | 395 | 209.5 | 1.89 |
| GCC1 | BC008902.2 | 306.5 | 208 | 1.47 |
| GMDS | NM_001500.1 | 374.5 | 136.5 | 2.74 |
| GPBP1L1 | NM_021639.2 | 397.5 | 180.5 | 2.20 |
| GRB7 | NM_001030002.1 | 335 | 202 | 1.66 |
| GTPBP10 | BC021573.1 | 356 | 195 | 1.83 |
| GTSF1 | NM_144594.1 | 233 | 183.5 | 1.27 |
| GUCD1 | BC070109.1 | 222 | 194.5 | 1.14 |
| HBS1L | NM_006620.2 | 419 | 262 | 1.60 |
| HMGN1 | BC070154.1 | 299.5 | 195.5 | 1.53 |
| HMGN3 | NM_138730.1 | 561 | 280.5 | 2.00 |
| HNRNPA1 | NM_002136.1 | 328.5 | 199.5 | 1.65 |
| HOXB6 | NM_156036.1 | 436.5 | 455.5 | 0.96 |
| IKBIP | NM_201613.1 | 353 | 147.5 | 2.39 |
| IVD | BC017202.2 | 376 | 172 | 2.19 |
| KCNAB1 | NM_172160.1 | 185.5 | 195 | 0.95 |
| KCTD18 | BC059366.1 | 361.5 | 96.5 | 3.75 |
| KIAA0020 | NM_014878.2 | 395 | 152 | 2.60 |
| KIAA0930 | NM_001009880.1 | 466.5 | 262 | 1.78 |
| LAMC1 | BC015586.2 | 796.5 | 336.5 | 2.37 |
| LARP4 | NM_199190.1 | 1161 | 304.5 | 3.81 |
| LCE3D | NM_032563.1 | 384.5 | 250.5 | 1.53 |
| LENG1 | NM_024316.1 | 451.5 | 252 | 1.79 |
| LGALS2 | NM_006498.1 | 402.5 | 111.5 | 3.61 |
| LGALS8-AS1 | BC014452.1 | 503 | 274.5 | 1.83 |
| LSM4 | NM_012321.1 | 334.5 | 177 | 1.89 |
| LYRM2 | NM_020466.3 | 329.5 | 133.5 | 2.47 |
| MAGEB1 | NM_002363.1 | 480 | 177 | 2.71 |
| MAGEB4 | BC032852.2 | 482 | 226 | 2.13 |
| MAP2 | NM_031845.1 | 1166 | 533.5 | 2.19 |
| MAPKAP5 | NM_003668.2 | 537.5 | 257 | 2.09 |
| MAPKAPK3 | BC001662.1 | 454.5 | 297 | 1.53 |
| MAPKAPK5 | NM_003668.2 | 393.5 | 221 | 1.78 |
| MEF2D | BC054520.1 | 336 | 102 | 3.29 |
| METAP2 | NM_006838.1 | 268.5 | 186 | 1.44 |
| MORF4L1 | NM_006791.1 | 323.5 | 155 | 2.09 |
| MRPL19 | NM_014763.2 | 496 | 296.5 | 1.67 |
| MTFR2 | NM_138419.1 | 324.5 | 191.5 | 1.69 |
| MTG1 | BC026039.1 | 460 | 189 | 2.43 |
| MTHFD2 | BC017054.1 | 369 | 132.5 | 2.78 |
| NECAP1 | NM_015509.2 | 351 | 105 | 3.34 |
| NEK7 | NM_133494.1 | 334 | 218.5 | 1.53 |
| NOL7 | NM_016167.3 | 355 | 210 | 1.69 |
| NOP16 | BC040106.1 | 369 | 206.5 | 1.79 |
| NSRP1 | NM_032141.1 | 479 | 216 | 2.22 |
| NUP50 | NM_007172.2 | 369.5 | 138 | 2.68 |
| ODF2L | BC009779.1 | 168.5 | 179.5 | 0.94 |
| ODF3L2 | BC104468.1 | 372.5 | 202 | 1.84 |
| PADI4 | NM_012387.1 | 362 | 202 | 1.79 |
| PAK4 | NM_005884.2 | 501 | 247 | 2.03 |
| PAPSS2 | BC009894.2 | 372.5 | 150.5 | 2.48 |
| PCK2 | BC001454.1 | 329.5 | 252 | 1.31 |
| PCLO | BC001304.1 | 347 | 395 | 0.88 |
| PCLO | BC001304.1 | 236 | 188 | 1.26 |
| PCTK1 | NM_033019.1 | 327.5 | 100.5 | 3.26 |
| PDAP1 | NM_014891.1 | 365 | 250 | 1.46 |
| PHF20L1 | NM_198513.1 | 649 | 373 | 1.74 |
| PHYH | NM_006214.2 | 348 | 213.5 | 1.63 |
| PICALM | NM_001008660.1 | 387.5 | 194.5 | 1.99 |
| PLEKHO2 | BC008744.2 | 371.5 | 128 | 2.90 |
| PLK1 | NM_005030.2 | 444 | 304 | 1.46 |
| PLK1 | BC002369.1 | 469 | 321 | 1.46 |
| POMZP3 | NM_152992.1 | 391 | 158 | 2.47 |
| POU5F1 | BC020712.1 | 276 | 193 | 1.43 |
| PPP1R14A | NM_033256.1 | 384 | 150 | 2.56 |
| PPP1R8 | NM_014110.3 | 319.5 | 185 | 1.73 |
| PRKRIP1 | BC014298.1 | 496 | 259.5 | 1.91 |
| PRR15 | NM_175887.2 | 565 | 300 | 1.88 |
| PSIP | BC033817.1 | 590 | 169.5 | 3.48 |
| PSMD4 | NM_153822.1 | 589 | 334.5 | 1.76 |
| PSMG1 | NM_003720.1 | 373 | 194 | 1.92 |
| PSRC1 | NM_032636.2 | 465.5 | 205.5 | 2.27 |
| PTPN6 | NM_080548.1 | 362.5 | 192 | 1.89 |
| PTPN7 | NM_080588.1 | 436.5 | 245 | 1.78 |
| PTRH2 | NM_016077.1 | 384 | 144.5 | 2.66 |
| PYROXD1 | NM_024854.2 | 464 | 141.5 | 3.28 |
| RAB11A | NM_004663.4 | 342.5 | 139.5 | 2.46 |
| RAB28 | BC035054.2 | 247 | 201.5 | 1.23 |
| RAD51AP1 | BC016330.1 | 325.5 | 162.5 | 2.00 |
| RALGPS1 | BC033708.1 | 373 | 266 | 1.40 |
| RGS3 | NM_134427.1 | 337 | 188.5 | 1.79 |
| RGS3 | BC019039.2 | 296 | 188 | 1.57 |
| RIT1 | NM_006912.3 | 292.5 | 193.5 | 1.51 |
| RIT2 | BC018060.1 | 352.5 | 267.5 | 1.32 |
| RND3 | NM_005168.3 | 568 | 141.5 | 4.01 |
| RNF4 | NM_002938.2 | 357.5 | 182.5 | 1.96 |
| RPL22 | NM_000983.3 | 666 | 321 | 2.07 |
| RPL35 | BC010919.1 | 415.5 | 210 | 1.98 |
| RPL39L | NM_052969.1 | 576.5 | 342 | 1.69 |
| RTF1 | NM_015138.2 | 334 | 105 | 3.18 |
| RWDD2B | NM_016940.1 | 550.5 | 232.5 | 2.37 |
| SAP18 | NM_005870.3 | 391 | 133.5 | 2.93 |
| SARNP | BC093051.1 | 328.5 | 201.5 | 1.63 |
| SARNP | NM_033082.1 | 310.5 | 186 | 1.67 |
| SCEL | BC047536.1 | 482 | 221.5 | 2.18 |
| SCYL1 | BC009967.1 | 279 | 315 | 0.89 |
| SF1 | NM_201998.1 | 355 | 216.5 | 1.64 |
| SGK2 | BC014037.1 | 341 | 170 | 2.01 |
| SH2D2A | NM_003975.1 | 283 | 194 | 1.46 |
| SHC3 | NM_016848.2 | 448.5 | 257.5 | 1.74 |
| SLAIN2 | BC031691.2 | 351.5 | 225.5 | 1.56 |
| SMAGP | NM_020467.2 | 538 | 305 | 1.76 |
| SMIM11 | BC015596.1 | 365 | 138 | 2.64 |
| SMNDC1 | BC011234.1 | 502.5 | 295 | 1.70 |
| SMTNL2 | NM_198501.1 | 324 | 151.5 | 2.14 |
| SNX9 | NM_016224.3 | 414 | 178.5 | 2.32 |
| SPATA20 | NM_022827.2 | 329.5 | 172.5 | 1.91 |
| SPATS2 | BC048299.1 | 495 | 257 | 1.93 |
| SRP19 | NM_003135.1 | 488 | 185 | 2.64 |
| SRP19 | BC010947.1 | 520.5 | 297 | 1.75 |
| SRSF5 | NM_006925.2 | 334 | 172 | 1.94 |
| STON1 | NM_006873.2 | 348 | 126.5 | 2.75 |
| SUDS3 | BC093990.1 | 602 | 330 | 1.82 |
| SULT1B1 | NM_014465.2 | 345 | 93 | 3.71 |
| SYK | NM_003177.3 | 454.5 | 263 | 1.73 |
| SYTL2 | NM_032943.2 | 369.5 | 183 | 2.02 |
| TACO1 | NM_016360.1 | 381.5 | 141 | 2.71 |
| TARP | NM_001003799.1 | 384 | 248.5 | 1.55 |
| TCEAL2 | NM_080390.3 | 676.5 | 324 | 2.09 |
| TEX33 | NM_178552.2 | 324 | 329 | 0.98 |
| TGIF2LX | NM_138960.3 | 376 | 151.5 | 2.48 |
| TGM1 | NM_000359.1 | 227 | 260.5 | 0.87 |
| TIGD1 | NM_145702.1 | 590.5 | 207.5 | 2.85 |
| TMA7 | NM_015933.1 | 333 | 187.5 | 1.78 |
| TRAPPC2 | NM_001011658.1 | 333 | 215.5 | 1.55 |
| TRIT1 | NM_017646.3 | 305 | 180.5 | 1.69 |
| TRUB1 | NM_139169.2 | 369 | 131 | 2.82 |
| UBE2B | BC001694.1 | 351 | 261.5 | 1.34 |
| UBE2E2 | NM_152653.1 | 430.5 | 253.5 | 1.70 |
| UBE2S | BC004236.2 | 499 | 366.5 | 1.36 |
| UBXN10 | NM_152376.2 | 382.5 | 227.5 | 1.68 |
| UFD1L | NM_005659.1 | 262 | 213.5 | 1.23 |
| USP4 | NM_003363.2 | 338 | 268.5 | 1.26 |
| VRK1 | NM_003384.1 | 530 | 375.5 | 1.41 |
| WHSC1 | NM_133336.1 | 344.5 | 205 | 1.68 |
| WHSC2 | NM_005663.2 | 721 | 212 | 3.40 |
| WIGB | NM_032345.1 | 547 | 170 | 3.22 |
| WIPF1 | BC002914.1 | 777.5 | 332.5 | 2.34 |
| XIAP | NM_001167.2 | 601 | 258.5 | 2.32 |
| ZFAND5 | NM_006007.1 | 375 | 133 | 2.82 |
| ZMYND11 | BC091489.1 | 353 | 196 | 1.80 |
| ZMYND19 | NM_138462.1 | 343 | 153.5 | 2.23 |
| ZNF174 | BC000876.1 | 387.5 | 128 | 3.03 |
| ZNF239 | BC026030.1 | 236 | 187.5 | 1.26 |
|  | XM_379114.1 | 376.5 | 248.5 | 1.52 |
|  | PV4202 | 337.5 | 218.5 | 1.54 |
|  | XM_378350.2 | 875.5 | 508 | 1.72 |
|  | XM_374177.2 | 616.5 | 337.5 | 1.83 |
|  | XM_086879.4 | 768.5 | 544 | 1.41 |
|  | BC031228.1 | 340.5 | 196 | 1.74 |
|  | XM_378514.1 | 426 | 237.5 | 1.79 |
|  | XM_373800.2 | 241 | 207 | 1.16 |
|  | NM_017692.1 | 276 | 204.5 | 1.35 |
|  |  |  |  |  |
