# Supplementary material for: Sorcin Links Calcium Signaling to Vesicle Trafficking, Regulates Polo-Like Kinase 1 and Is Necessary for Mitosis
Source: PLoS One. 2014 Jan 10;9(1):e85438. doi: 10.1371/journal.pone.0085438 (PMC3888430; doi:10.1371/journal.pone.0085438)
Supplement: Table S3 — Proteins interacting with sorcin and identified in the midbody. (DOCX) [file pone.0085438.s007.docx]

**Table S3.**

Proteins interacting with sorcin and identified in the midbody, according to:

Skop AR, Liu H, Yates J, 3rd, Meyer BJ, Heald R (2004) Dissection of the mammalian midbody proteome reveals conserved cytokinesis mechanisms. Science 305: 61-66.

Chen TC, Lee SA, Hong TM, Shih JY, Lai JM, et al. (2009) From midbody protein-protein interaction network construction to novel regulators in cytokinesis. J Proteome Res 8: 4943-4953.

| **ANXA11**: Annexin 11 | A member of the annexin family, a group of calcium-dependent phospholipid-binding proteins. Binds specifically to calcyclin in a calcium-dependent manner. Predominantly nuclear. Required for midbody formation and completion of the terminal phase of cytokinesis. |
| --- | --- |
| **APEX1**: APEX nuclease (multifunctional DNA repair enzyme) 1 | Apurinic/apyrimidinic (AP) sites occur frequently in DNA molecules by spontaneous hydrolysis, by DNA damaging agents or by DNA glycosylases that remove specific abnormal bases. AP sites are pre-mutagenic lesions that can prevent normal DNA replication so the cell contains systems to identify and repair such sites. Class II AP endonucleases cleave the phosphodiester backbone 5' to the AP site. This gene encodes the major AP endonuclease in human cells. |
| **AURKA**: Aurora kinase A | A member of the aurora kinase subfamily of serine/threonine kinases. These kinases participate in the regulation of segregation of chromosomes during mitosis and meiosis through association with microtubules. AURKA associates with the centrosome and the spindle microtubules during mitosis and plays a critical role in various mitotic events including the establishment of mitotic spindle, centrosome duplication, centrosome separation as well as maturation, chromosomal alignment, spindle assembly checkpoint, and cytokinesis. Required for initial activation of CDK1 at centrosomes. Phosphorylates numerous target proteins. Found at the centrosome in interphase cells and at the spindle poles in mitosis. This gene may play a role in tumor development and progression. |
| **AURKB**: Aurora kinase B | A member of the aurora kinase subfamily of serine/threonine kinases. These kinases participate in the regulation of segregation of chromosomes during mitosis and meiosis through association with microtubules. AURKB is a component of the chromosomal passenger complex (CPC), a complex that acts as a key regulator of mitosis. The CPC complex has essential functions at the centromere in ensuring correct chromosome alignment and segregation and is required for chromatin-induced microtubule stabilization and spindle assembly. Involved in the bipolar attachment of spindle microtubules to kinetochores and is a key regulator for the onset of cytokinesis during mitosis. Required for central/midzone spindle assembly and cleavage furrow formation. |
| **CPNE1**: Copine 1 | Calcium-dependent membrane-binding proteins may regulate molecular events at the interface of the cell membrane and cytoplasm. This calcium-dependent protein also contains two N-terminal type II C2 domains and an integrin A domain-like sequence in the C-terminus. However, the encoded protein does not contain a predicted signal sequence or transmembrane domains. This protein has a broad tissue distribution and it may function in membrane trafficking. |
| **CSNK2A1**: Casein kinase 2, alpha 1 polypeptide | Casein kinase II is a serine/threonine protein kinase that phosphorylates acidic proteins such as casein. The kinase exists as a tetramer and is composed of an alpha, an alpha-prime, and two beta subunits. The alpha subunits contain the catalytic activity while the beta subunits undergo autophosphorylation. The protein encoded by this gene represents the alpha subunit. |
| **CSNK2A2**: Casein kinase 2, alpha prime polypeptide | Casein kinases are operationally defined by their preferential utilization of acidic proteins such as caseins as substrates. The alpha and alpha' chains contain the catalytic site. Participates in Wnt signaling. CK2 phosphorylates 'Ser-392' of p53/TP53 following UV irradiation. Interacts with calmodulin. |
| **CTNND1**: Catenin (cadherin-associated protein), delta 1 | A member of the Armadillo protein family, which function in adhesion between cells and signal transduction. |
| **HNRNPA1**: Heterogeneous nuclear ribonucleoprotein A1 | This gene belongs to the A/B subfamily of ubiquitously expressed heterogeneous nuclear ribonucleoproteins (hnRNPs). The hnRNPs are RNA binding proteins and they complex with heterogeneous nuclear RNA (hnRNA). These proteins are associated with pre-mRNAs in the nucleus and appear to influence pre-mRNA processing and other aspects of mRNA metabolism and transport. While all of the hnRNPs are present in the nucleus, some seem to shuttle between the nucleus and the cytoplasm. The hnRNP proteins have distinct nucleic acid binding properties. This protein has two repeats of quasi-RRM domains that bind to RNAs. It is one of the most abundant core proteins of hnRNP complexes and it is localized to the nucleoplasm. This protein, along with other hnRNP proteins, is exported from the nucleus, probably bound to mRNA, and is immediately re-imported. Its M9 domain acts as both a nuclear localization and nuclear export signal. The protein is involved in the packaging of pre-mRNA into hnRNP particles, transport of poly A+ mRNA from the nucleus to the cytoplasm, and may modulate splice site selection. |
| **METAP2**: Methionyl aminopeptidase 2 | A member of the methionyl aminopeptidase family that binds 2 cobalt or manganese ions. This protein functions both by protecting the alpha subunit of eukaryotic initiation factor 2 from inhibitory phosphorylation and by removing the amino-terminal methionine residue from nascent protein. Increased expression of this gene is associated with various forms of cancer and the anti-cancer drugs fumagillin and ovalicin inhibit the protein by irreversibly binding to its active site. |
| **MTHFD2**: Methylenetetrahydrofolate dehydrogenase (NADP+ dependent) 2, methenyltetrahydrofolate cyclohydrolase | A nuclear-encoded mitochondrial bifunctional enzyme with methylenetetrahydrofolate dehydrogenase and methenyltetrahydrofolate cyclohydrolase activities. The enzyme functions as a homodimer and is unique in its absolute requirement for magnesium and inorganic phosphate. Formation of the enzyme-magnesium complex allows binding of NAD. |
| **PCLO:** Piccolo (presynaptic cytomatrix protein) | It is part of the presynaptic cytoskeletal matrix, which is involved in establishing active synaptic zones and in synaptic vesicle trafficking. Variations in this gene have been associated with bipolar disorder and major depressive disorder. |
| **PICALM**: Phosphatidylinositol binding clathrin assembly protein | Assembly protein recruiting clathrin and adaptor protein complex 2 (AP2) to cell membranes at sites of coated-pit formation and clathrin-vesicle assembly. May be required to determine the amount of membrane to be recycled, possibly by regulating the size of the clathrin cage. Involved in AP2-dependent clathrin-mediated endocytosis at the neuromuscular junction. |
| **PLK1**: Polo-like kinase 1 | Serine/threonine-protein kinase that performs several important functions throughout M phase of the cell cycle, including the regulation of centrosome maturation and spindle assembly, the removal of cohesins from chromosome arms, the inactivation of anaphase-promoting complex/cyclosome (APC/C) inhibitors, and the regulation of mitotic exit and cytokinesis. Plays a key role in centrosome functions and the assembly of bipolar spindles, and promotes targeting of the gamma-tubulin ring complex (gTuRC) to the centrosome, an important step for spindle formation. Involved in mitosis exit and cytokinesis by phosphorylating many target proteins. Plays a central role in G2/M transition of mitotic cell cycle by phosphorylating CCNB1, CDC25C, FOXM1, MLF1IP, PKMYT1/MYT1, PPP1R12A/MYPT1 and WEE1. Part of a regulatory circuit that promotes the activation of CDK1 by phosphorylating the positive regulator CDC25C and inhibiting the negative regulators WEE1 and PKMYT1/MYT1. Mediates phosphorylation of cyclin-B1 on centrosomes in prophase. Phosphorylates key mitotic transcription regulators. Involved in kinetochore functions and sister chromatide cohesion. PLK1 is high on non-attached kinetochores suggesting a role of PLK1 in kinetochore attachment or in spindle assembly checkpoint (SAC) regulation. Regulates the dissociation of cohesin from chromosomes. Phosphorylates proteins determining their spindle pole localization; plays a role in regulating centriole cohesion function. Mediates phosphorylation of FBXO5/EMI1, a negative regulator of the APC/C complex during prophase, leading to FBXO5/EMI1 ubiquitination and degradation by the proteasome. Acts as a negative regulator of p53 family members. Phosphorylates the transactivation domain of the transcription factor p73/TP73, leading to inhibit p73/TP73-mediated transcriptional activation and pro-apoptotic functions. Phosphorylates BORA, and thereby promotes the degradation of BORA. Contributes to the regulation of AURKA function. Also required for recovery after DNA damage checkpoint and entry into mitosis. |
| **PPP1R8**: protein phosphatase 1, regulatory subunit 8 | This gene, through alternative splicing, encodes three different isoforms. Two of the protein isoforms encoded by this gene are specific inhibitors of type 1 serine/threonine protein phosphatases and can bind but not cleave RNA. The third protein isoform lacks the phosphatase inhibitory function but is a single-strand endoribonuclease comparable to RNase E of *E. coli*. This isoform requires magnesium for its function and cleaves specific sites in A+U-rich regions of RNA. |
| **PSRC1**: Proline/serine-rich coiled-coil 1 | A proline-rich protein. This gene is regulated by p53 and may participate in p53-mediated growth suppression. The encoded protein may function as a microtubule destabilizing protein that controls spindle dynamics and mitotic progression by recruiting and regulating microtubule depolymerases. At least one genetic variation in this gene has been associated with decreased serum levels of low-density lipoprotein cholesterol. |
| **RPL22**: Ribosomal protein L22 | Ribosomes, the organelles that catalyze protein synthesis, consist of a small 40S subunit and a large 60S subunit. Together these subunits are composed of 4 RNA species and approximately 80 structurally distinct proteins. This is a cytoplasmic ribosomal protein that is a component of the 60S subunit. The protein belongs to the L22E family of ribosomal proteins. The protein can bind specifically to Epstein-Barr virus-encoded RNAs (EBERs) 1 and 2. The mouse protein has been shown to be capable of binding to heparin. Transcript variants utilizing alternative polyA signals exist. As is typical for genes encoding ribosomal proteins, there are multiple processed pseudogenes of this gene dispersed through the genome. |
| **SCYL1**: SCY1-like 1 (S. cerevisiae) | A transcriptional regulator belonging to the SCY1-like family of kinase-like proteins. The protein has a divergent N-terminal kinase domain that is thought to be catalytically inactive, and can bind specific DNA sequences through its C-terminal domain. It activates transcription of the telomerase reverse transcriptase and DNA polymerase beta genes. The protein has been localized to the nucleus, and also to the cytoplasm and centrosomes during mitosis. |
| **SF1**: Splicing factor 1 | A nuclear pre-mRNA splicing factor. The protein specifically recognizes the intron branch point sequence and is required for the early stages of spliceosome assembly. |
| **USP4**: Ubiquitin specific peptidase 4 (proto-oncogene) | A protease that deubiquitinates target proteins such as ADORA2A and TRIM21. The protein shuttles between the nucleus and cytoplasm and is involved in maintaining operational fidelity in the endoplasmic reticulum. |
